# Aberrant activated Notch1 promotes prostate enlargement driven by androgen signaling via disrupting mitochondrial function in mouse

**DOI:** 10.1007/s00018-024-05143-0

**Published:** 2024-03-28

**Authors:** Jin-Wen Kang, Jia-Peng He, Ying-Nan Liu, Yu Zhang, Shan-Shan Song, Qi-Xin Xu, Shu-Wen Wei, Lei Lu, Xiang-Qi Meng, Lin Xu, Bin Guo, Ren-Wei Su

**Affiliations:** 1 College of Veterinary Medicine, South China Agricultural University, Guangzhou, PR China; 2 Department of General Surgery, The Sixth Affiliated Hospital, Sun Yat-sen University, Guangzhou, PR China; 3 College of Sports and Human Science, Harbin Sport University, Harbin, PR China; 4 College of Veterinary Medicine, Jilin University, Changchun, PR China; 5 Key Laboratory of Animal Vaccine Development, Ministry of Agriculture, Guangzhou, PR China

**Keywords:** Notch1 signaling, Mouse, Prostate enlargement, DHT, ROS

## Abstract

The prostate is a vital accessory gonad in the mammalian male reproductive system. With the ever-increasing proportion of the population over 60 years of age worldwide, the incidence of prostate diseases, such as benign prostatic hyperplasia (BPH) and prostate cancer (PCa), is on the rise and is gradually becoming a significant medical problem globally. The notch signaling pathway is essential in regulating prostate early development. However, the potential regulatory mechanism of Notch signaling in prostatic enlargement and hyperplasia remains unclear. In this study, we proved that overactivation of Notch1 signaling in mouse prostatic epithelial cells (*OEx*) led to prostatic enlargement via enhancing proliferation and inhibiting apoptosis of prostatic epithelial cells. Further study showed that N1ICD/RBPJ directly up-regulated the androgen receptor (AR) and enhanced prostatic sensitivity to androgens. Hyper-proliferation was not found in orchidectomized *OEx* mice without androgen supply but was observed after Dihydrotestosterone (DHT) supplementation. Our data showed that the number of mitochondrion in prostatic epithelial cells of *OEx* mice was increased, but the mitochondrial function was impaired, and the essential activity of the mitochondrial respiratory electron transport chain was significantly weakened. Disordered mitochondrial number and metabolic function further resulted in excessive accumulation of reactive oxygen species (ROS). Importantly, anti-oxidant N-Acetyl-L-Cysteine (NAC) therapy could alleviate prostatic hyperplasia caused by the over-activation of Notch1 signaling. Furthermore, we observed the incremental Notch signaling activity in progenitor-like club cells in the scRNA-seq data set of human BPH patients. Moreover, the increased number of TROP2^+^ progenitors and Club cells was also confirmed in our *OEx* mice. In conclusion, our study revealed that over-activated Notch1 signaling induces prostatic enlargement by increasing androgen receptor sensitivity, disrupting cellular mitochondrial metabolism, increasing ROS, and a higher number of progenitor cells, all of which can be effectively rescued by NAC treatment.

**Supplementary Information:**

The online version contains supplementary material available at 10.1007/s00018-024-05143-0.

## Introduction

As a vital accessory gonad of the male reproductive system, the primary physiological function of the prostate is to produce and secrete prostatic fluid, an essential component of semen [1]. In anatomy, the human prostate includes the transition, central, and peripheral zones, and the mouse prostate can be divided into anterior, ventral, lateral, and dorsal lobes [2]. In humans, the prostate sits near the bladder neck and wraps around the urethra to form the proximal urethra wall, which controls urination. Prostate growth continuously in adults, the volume increases by approximately 2.5% annually, according to a 4.3 years following-up study [3]. The enlarged prostate compresses the urethra, causing intense stimulation and obstruction to the urethra, resulting in dysuria, which is in terms of benign prostatic hyperplasia (BPH) [4]. Approximately 50% of men > 50 years of age suffer from BPH and the associated lower urinary tract symptoms (LUTS), with this number increasing to > 80% when they reach their 80s and older [5]. The BPH leads to severe health and economic consequences for patients, families, and society, which have rapidly increased in recent years because of the rapidly increased aging population globally [6]. Another prostatic disease associated with hyperplasia is prostate cancer (PCa), the second leading cause of death from cancer in men [7]. Autopsy studies hint that as many as 59% of men aged 80 years and older may harbor carcinomas in their prostates, most of which go unrecognized [8]. To date, the association between BPH and PCa is still a controversial issue. However, the two diseases share traits and risk factors such as age, hormone response, prostate inflammation, and metabolic disruption [9], but they differ in terms of histology and localization: the majority of BPH and PCa arise from the transition and peripheral zone of human prostate, respectively [5, 10].

The prostate is an androgen-dependent organ, and the androgen signaling pathway dominantly regulates the development and function of the prostate, the size of the prostate shrinks after castration [11]. Androgens play their roles by directly binding to the nuclear receptor androgen receptor (AR), which is highly expressed in prostate epithelial cells, and binds to the promoter region of target genes to activate or inhibit transcription [12]. Many studies have shown that androgen/androgen receptor (AR) signaling plays a crucial role in prostatic hyperplasia [13, 14]. Among all the androgens, dihydrotestosterone (DHT) is the most active form and the principal androgen in the prostate, accounting for 90% of total prostatic androgen [15]. The intraprostatic DHT levels remain high with aging, even the plasma testosterone drops [15]. This discrepancy is attributed to the conversion of testosterone to DHT in the prostate gland by 5α -reductase [16]. Previous studies have shown that increased activity of this enzyme results in an increased DHT/testosterone ratio in older men, further promoting prostatic cell hyper-proliferation and resulting in hyperplasia [17, 18]. Large, randomized trials also show the roles of 5α-reductase inhibitors in improving BPH-associated LUTS and prostate cancer prevention [9]. Studies suggest that DHT regulates the proliferation and death balance by controlling the production of different growth factors such as KGF, EGF, IGFs, and TGFβ, unbalance of this mechanism leads to BPH [5].

The Notch signaling pathway is evolutionarily conserved in multicellular organisms and plays regulatory roles in cell fate determination and maintenance of tissue homeostasis during biological development [19]. Canonical Notch signaling is initiated after the Notch transmembrane receptor (Notch1-Notch4) interacts with cell-binding ligands (δ like 1, 3, or 4 or serrated 1 or 2), a process that involves the Notch cleavage cascade by ADAM proteases and γ-secretase [20]. The expression of Notch1 changes dynamically during prostate development. *Notch1* mRNA is highly expressed in embryonic and postnatal prostate epithelial cells and is subsequently down-regulated as the prostate matures [21]. Dysregulation of Notch signaling leads to various types of cancer in different tissues, such as breast cancer, gastric cancer, and pancreatic cancer [22], and elevated Notch1 expression has been observed in malignant prostatic epithelial cells of primary and metastatic tumors [23, 24]. However, the role of Notch1 signaling in DHT-dependent enlargement of the prostate is rarely studied.

Herein, we proved that the constitutive over-activation of Notch1 signaling led to DHT-dependent prostatic enlargement by establishing a prostatic epithelial specifically over-activating Notch1 signaling pathway (*OEx*) mouse model. Our study showed that over-activated Notch1 signaling in the mouse prostate promoted epithelial cell proliferation and inhibited apoptosis via increasing reactive oxygen species (ROS) levels. In addition, anti-oxidant NAC could effectively inhibit prostate enlargement induced by over-activating Notch1 signaling in our mouse model. At last, we found that the Notch and androgen signaling were increased in a sub-population of prostatic epithelial cells in humans with BPH by analyzing single-cell sequencing data, and the number of TROP2^+^ progenitors was increased in the *OEx* prostate.

## Materials and methods

### Animals

*Pbsn-Cre* mice, *Rosa26*^*N1ICD/N1ICD*^ mice, and *Rosa26*^*mTmG/mTmG*^ mice were purchased from The Jackson Laboratory (Bar Harbor, ME, USA). All mice were housed in an SPF facility with a 14-hour light/10-hour dark cycle. *Pbsn-Cre* male mice were bred to *Rosa26*^*N1ICD/N1ICD*^ or *Rosa26*^*mTmG/mTmG*^ female mice to generate *Pbsn*^*Cre*^*Rosa26*^*N1ICD/+*^ or *Pbsn*^*Cre*^*Rosa26*^*mTmG/+*^ mice, respectively. Notch1 intracellular domain (*N1ICD*) was specifically overexpressed in the prostatic epithelium of *Pbsn*^*Cre*^*Rosa26*^*N1ICD/+*^ (*OEx*) male mice. *Rosa26*^*N1ICD/+*^ mice were used as controls (*Ctrl*). Male mice were sacrificed, and the anterior prostates (AP) were collected at ages 8, 12, 16, and 20 weeks. The AP was weighted and then fixed in 4% paraformaldehyde (PFA) for histological analysis or snap-frozen in liquid nitrogen for further use. Metabolic cages (Tecniplast GmbH, Hohenpeißenberg, Germany) were used to quantify 24-h urine and drink volumes of both *Ctrl* and *OEx* mice at 20 weeks of age.

### Orchidectomy model and drug treatment

Males were orchidectomized (ORX) as described by Antonia Sophocleous [25]. Briefly, the 6-week-old male mice were anesthetized, an incision was made in the abdomen, a single ligature was performed around the vas deferens and blood vessels, and then the testis by gently severing blood vessels with small scissors. After 3 weeks of rest, male mice were subcutaneously injected with DHT (500 µg/kg body weight, Sigma) or sesame oil for 3 days. Regarding the NAC treatment, mice were ORX, with 8 weeks of rest, intraperitoneally treated with N-acetyl-L-cysteine (NAC, 75 µg/kg body weight; Beyotime) for 2 days, and then NAC + DHT treatment for another 3 days, the anterior prostates were collected 24 h after the last treatment (Fig. S4). Another set of mice was *i.p.* injected with 0.1 mL 1.5% H_2_O_2_ per day for 2 weeks.

### Measurement of serum T and DHT levels

Mice were anesthetized, and serum was collected from the venous sinus. Serum T and DHT levels were measured by enzyme immunoassays performed at Shanghai Yanhui Biotechnology Co., LTD. The R-value of linear regression of standard substance and the expected concentration is greater than or equal to 0.9900. This assay has a sensitivity of 0.1 ng/mL, with intra- and interassay coefficients of variation of 15%.

### Histological and Immuno-staining

Frozen prostatic tissues of *Pbsn*^*Cre*^*Rosa26*^*mTmG/+*^ or *Rosa26*^*mTmG/+*^ mice were sectioned to 10 μm and then fixed in 4% PFA for 1 h at room temperature. All sections were counterstained with DAPI (4′,6-diamidino-2-phenylindole, Thermo Fisher). Images were captured using a confocal microscope (Leica, TCS SP8, Germany). 4% PFA-fixed, paraffin-embedded prostatic tissues were sectioned to 5 μm. After deparaffinization and hydration, sections were stained with H&E for histological analysis. For immunohistochemistry and immunofluorescence, sections were subjected to antigen retrieval in a pH 6.0 Sodium Citrate buffer, followed by hydrogen peroxide treatment to inactive endogenous peroxidase. After blocking with 10% Normal Goat Serum (NGS), the sections were incubated with primary and biotin or fluorescence-labeled secondary antibodies, respectively. For immunohistochemistry, diaminobenzidine (ORIGENE, Beijing, China) was used to visualize antigens, followed by counterstaining with hematoxylin. DAPI was used for counterstaining for immunofluorescence, and Images were captured using a confocal microscope (Leica, TCS SP8, Germany). All primary antibodies used in this study are listed in Table S1.

### Digital HSCORE analysis

Digital HSCORE analysis for AR protein was performed using image analysis software (Image J, National Institutes of Health, Bethesda, Maryland, USA) as previously described [26, 27].

### Analysis of ROS level and mitochondrial complex activity analysis

The ROS intensity and mitochondrial complex activity were performed using the Tissue reactive oxygen species kit (ChemicalBook, Beijing, China) or the Mitochondrial complex kit (KTB1850, KTB1870, KTB1880, Abbkine) according to the manufacturer’s protocol, respectively. Briefly, the fresh AP tissues were digested, and the cellular contents of AP were extracted. After several times gradient centrifugation, the reaction reagent was added to the extraction solution. Finally, the ROS intensity values of the fluorescence spectrophotometer with an emission wavelength of 488 nm and absorption wavelength of 520 nm were read. The values of the microplate reader at the absorbance of 340 nm (Complex I) or 550 nm (Complex III and IV) were read. Finally, perform data calculation and analysis according to product specifications.

### Cell culture and plasmid transfection

The human prostatic RWPE1 epithelial cells were cultured in DMEM/F12 (D2906, Sigma-Aldrich) supplemented with 10% FBS, and incubated at 37゜C in a humidified atmosphere of 95% air and 5% CO_2_. The DNA sequence encoding the intracellular domain of hNOTCH1 (N1ICD) was cloned into the pcDNA3.1 vector and named pcDNA3.1-N1ICD. The empty pcDNA3.1 was used as a control. The *AR* promoter sequence containing the RBPJ binding site (WT) or its mutant site (MUT) was inserted into the pGL3 vector and synthesized by SeyontinBIO-TECH CO.LTD, Guangzhou, China. The RWPE1 cells were seeded in 24-well plate, and 100 ng pcDNA3.1-N1ICD or pcDNA3.1, together with 700 ng WT-pGL3 or MUT-pGL3 were transfected using Lipofectamine 2000™ (Invitrogen) reagent with plasmids used according to the instructions of the manufacturer. Meanwhile, 100 ng of pRL-TK was co-transfected as an internal control.

### Dual luciferase assay

48 h after transfection, luciferase activity was evaluated by dual-luciferase reporter gene assay kit (#RG088M, Beyotime, Shanghai, China) according to the manufacturer’s instructions. Briefly, the cells were washed by HBSS twice and then lysed by lysis buffer, and then the activity of firefly and renilla luciferase was measured by GLOMAX 20/20 luminometer (Promega, Madison, USA).

### Western blot

Tissues were lysed in RIPA Lysis Buffer (Yamei, Shanghai, China), and proteins were quantified using Pierce™ BCA Protein Assay Reagent (Thermo, Carlsbad, USA). 10 µg total proteins were loaded on 10% SDS-PAGE gel and transferred onto a 0.45 μm nitrocellulose membrane. Blotting membranes were labeled with primary antibodies at 4゜C overnight after 1 h of blocking by 5% milk at room temperature. The membranes were then incubated with HRP-labeled secondary antibodies and enhanced chemiluminescence (ECL, Millipore, USA), respectively. At last, the signals were scanned by a Tanon-5200 imager (Tanon, Shanghai, China). All primary antibodies used in this study are listed in Table S1.

### RNA isolation and qRT-PCR

The prostatic secretions were cleaned to prevent poor RNA quality [28]. According to the manufacturer’s introduction, total RNA was extracted from tissues using a Trizol RNA reagent (Takara, Dalian, China). The RNAs were then reverse transcripted into cDNA using a HiSuperscript cDNA synthesis kit (Vazyme). qPCR was performed using an SYBR qPCR Master Mix kit (Vazyme) following the manufacturer’s protocol on the BIORAD-CFX96 Real-Time System (Bio-Rad, Hercules, CA, USA). The data were normalized by mouse *Rpl19* and analyzed using the ΔΔCt method. All qPCR primers of mice were listed in Table S2.

### RNA-Seq analysis

We collected APs from 20-week-old male mice for RNA-Seq analysis. We used the Trizol RNA Reagent (Takara, Dalian, China) to extract total RNA from the tissues, and the concentration and integrity of the RNA were measured with the ND-1000 Nanodrop and the Agilent 2100 TapeStation (Novogene Bioinformatic Technology, Beijing, China). The quality control parameters used in this study were: A260/A280 ratio ≥ 1.8, A260/A230 ratio ≥ 2.0, and RNA integrity number ≥ 8.0. The TruSeq RNA sample preparation kit (Illumina, San Diego, CA, USA) was used to generate cDNA libraries. High-throughput sequencing was run on an Illumina HiSeq 2500 system. Raw RNA-seq data were processed with an in-house computational pipeline. Differentially expressed genes were chosen based on fold change criteria > 2 and FDR (false discovery rate) < 0.05. The RNA-seq raw data were deposited in Gene Expression Omnibus (GEO) with the accession number GSE200565.

### GO and KEGG Enrichment analyses

GO and KEGG analysis was performed using the DAVID online tools [29]. The RNA-enriched genes were analyzed by using the GO database and KEGG database. The false discovery rate (FDR) cutoff was set at 0.05.

### scRNA-Seq Data Analysis

The single-cell RNA-seq data for benign prostatic hyperplasia and control were downloaded from the GEO database (GSE145928). Annotation information was provided by Dr. Strand DW [30]. Cells with less than 200 or greater than 6000 unique genes were excluded. The gene count matrix was normalized by SCTransform using default settings. Gene expression pattern in each cell type was visualized using the R package ggplot2.

### Statistics

Statistical analyses were conducted using a two-tailed Student’s t-test for those two groups’ analyses. And two-way analysis of variance (ANOVA) with Tukey’s multiple comparison test was used to determine significance in multiple-group experiments. All statistical analyses were performed using GraphPad Prism 8.0 (GraphPad Software). Data are shown as mean ± SEM. *P* < 0.05 was considered a statistical significance.

## Results

### Over-activated Notch1 signaling enlarges prostates in mice

**Fig. 1:**
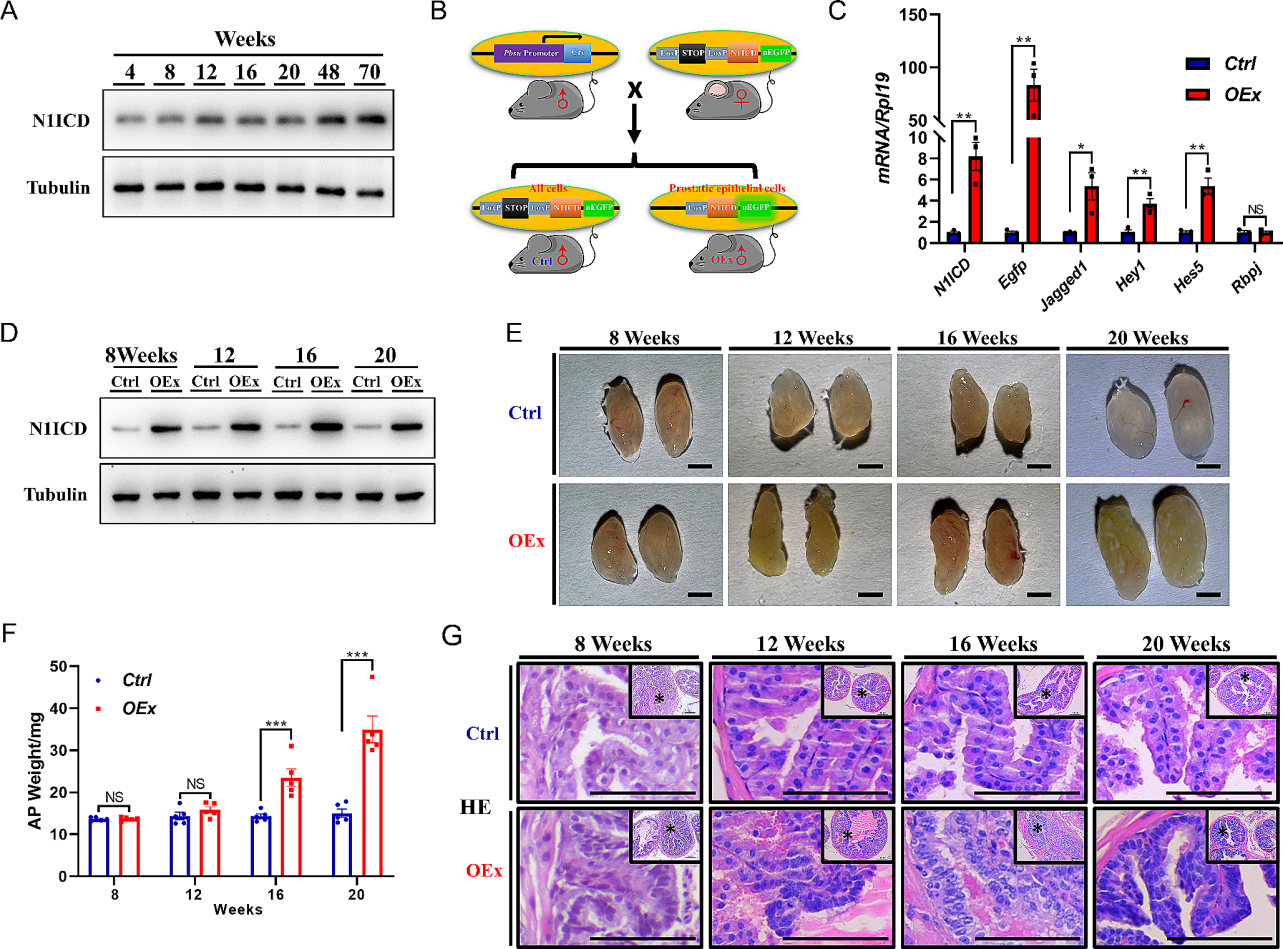
Prostatic enlargement in *OEx* male mice. (**A**) Western blot analysis of N1ICD protein level in AP at different ages. (**B**) Generation strategy of *Rosa26*^*N1ICD/+*^ (*Ctrl*) and *Pbsn-Cre Rosa26*^*N1ICD/+*^ (*OEx*) mice. (**C**) Expression of *N1ICD*, *Egfp*, *Jagged1*, *Hey1*, *Hes5*, and *Rbpj* mRNA in the prostate of *Ctrl* and *OEx* mice. Data are shown as mean ± SEM, *n* = 3 mice. (**D**) Comparison of the protein levels of N1ICD in *Ctrl* and *OEx* mice at 8, 12, 16, and 20 weeks of age. Morphology (**E**) and wet weight (**F**) of anterior prostate (AP) from *Ctrl* and *OEx* mice at 8, 12, 16 and 20 weeks of age. Data are shown as mean ± SEM, *n* = 5 mice. (**G**) Histological staining of AP from *Ctrl* and *OEx* mice. Scale bar: 2 mm in C, 100 μm in E; NS: No Significance; *p < 0.05; **p < 0.01; ***p < 0.001

To test if the activation of Notch signaling is associated with the growth of the prostate with age, we first detected the expression level of N1ICD (the activation status of Notch1) in the prostate of mice of different ages. The results showed that the expression level of N1ICD increased with age (Fig. 1A). To investigate the roles of high-level Notch1 signaling in mouse prostate, we generated a prostatic epithelium-specific Notch1 intracellular domain (*N1ICD*) over-expression mice model (*Pbsn-Cre Rosa*^*N1ICD/+*^, *OEx*), with the flow chart showing in Fig. 1B. The specific activity of *Pbsn-Cre* used in this study was confirmed by crossing with an mT/mG red-green double-fluorescent Cre-reporter mice, in which the Cre-activated cells express GFP while other cells are expressing RFP (Fig. S1A&B). The higher expression of both *N1ICD* and co-inserted *Egfp* mRNAs was detected in *OEx* mice compared to *Rosa*^*N1ICD/+*^ mice (*Ctrl*), suggesting the success of overexpressing *N1ICD* (Fig. 1C). Moreover, the increased expression of N1ICD protein and the mRNA levels of Notch signaling targets *Hey1* and *Hes5* indicated the functional over-activation of Notch signaling in the prostate of our mouse model (Fig. 1C&D). We then analyzed the prostate morphology of both *Ctrl* and *OEx* mice at different weeks of age. At 8 and 12 weeks, there was no significant difference in the size, weight, or histological morphology in *OEx* mice compared with *Ctrl* mice (Fig. 1E-G). However, at 16 and 20 weeks, the *OEx* mice showed an apparent increase in anterior prostate (AP) size and wet weight than the *Ctrl* mice (Fig. 1E&F). The density of AP epithelial cells was also significantly increased, and the cells were crowded, forming many convex nodules and clusters with a more complex overall structure (Fig. 1G). On the other hand, the size and morphology of other male reproductive organs, such as testis, epididymis, seminal vesicle, and vas deferens in *OEx* mice remained comparable with *Ctrl* mice (Fig. S2A&B). The 24-hour urine volume monitoring of *OEx* mice showed no significant difference with *Ctrl* mice, suggesting normal urination function in these mice (Fig. S2C).

**Fig. 2:**
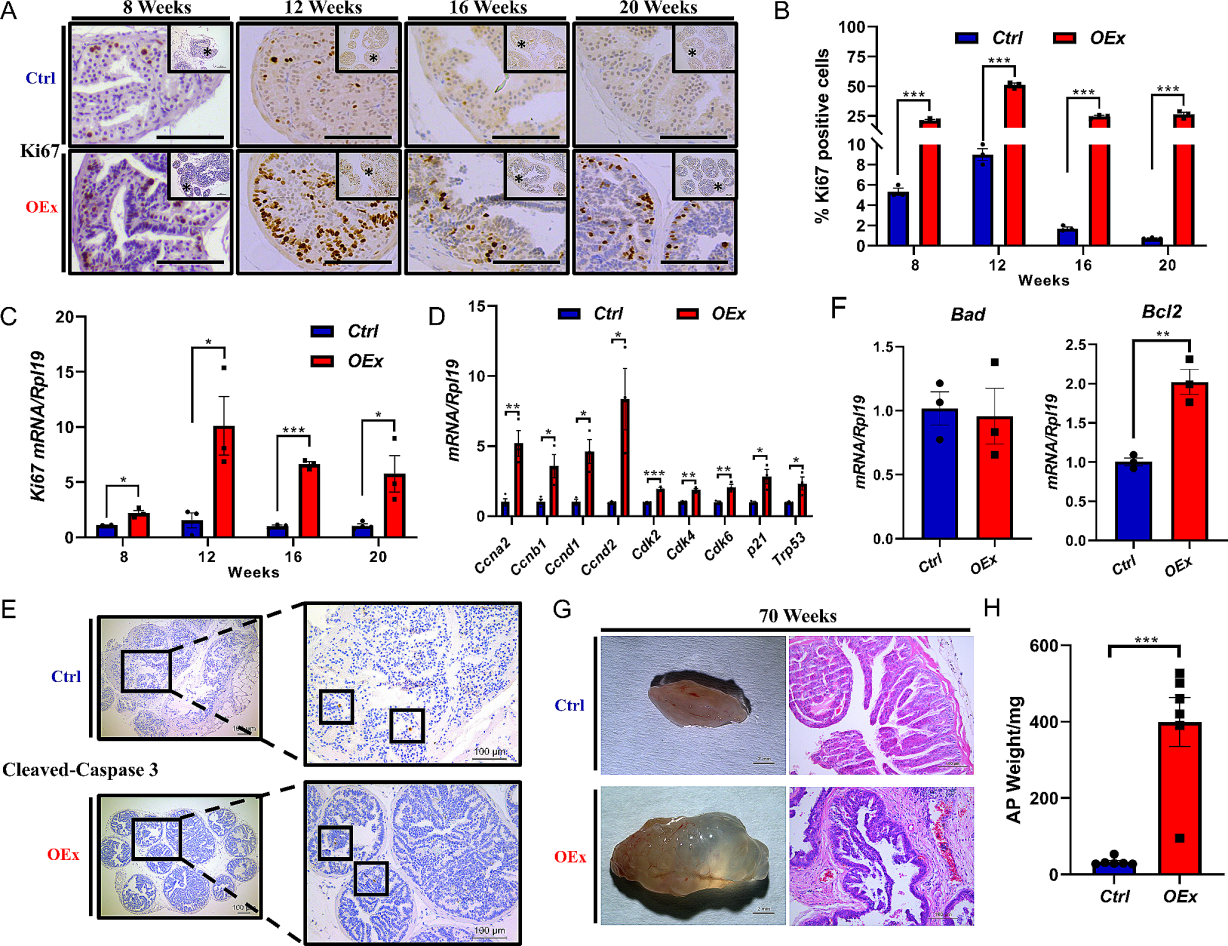
Proliferation and apoptosis statues in AP of *Ctrl* and *OEx* mice. (**A**) Immunostaining of Ki67 in AP from *Ctrl* and *OEx* mice at 8, 12, 16, and 20 weeks of age. The quantitative expression level of Ki67 protein is shown in (**B**). Mean ± SEM, *n* = 3. (**C**) qPCR detected mRNA levels of the *Ki67* gene in AP from *Ctrl* and *OEx* mice at 8, 12, 16, and 20 weeks of age. Mean ± SEM, *n* = 3. (**D**) qPCR detected expression of cell cycle regulators *CyclinA2*, *Cyclin B1*, *CyclinD1*, *CyclinD2*, *CDK2*, *CDK4*, and *CDK6* in AP from *Ctrl* and *OEx* mice at 20 weeks of age. Mean ± SEM, *n* = 3. (**E**) Immunostaining of cleaved Caspase-3 in AP from *Ctrl* and *OEx* mice at 20 weeks of age. (**F**) The expression of *Bad* and *Bcl2* mRNA was detected by qPCR in AP from *Ctrl* and *OEx* mice at 20 weeks of age. Mean ± SEM, *n* = 3. (**G**) Morphological and histological images of AP from *Ctrl* and *OEx* mice at 1.5 years of age. (**H**) The wet weight of AP from *Ctrl* and *OEx* mice at 20 weeks. Scale bar: 100 μm in (**A**). Data are shown as mean ± SEM, *n* = 6 mice. *P < 0.05; **P < 0.01; ***P < 0.001

To further confirm if the enlarged prostate in *OEx* mice was due to hyper-proliferation, we detected the immunosignal of Ki67, a cell proliferation marker. The results showed that the number of Ki67 positive epithelial cells in AP from *OEx* mice was significantly increased at all time points from 8 to 20 weeks, although there was no difference in AP wet weight between *OEx* and *Ctrl* mice at 8 and 12 weeks (Fig. 2A&B). The qPCR results also confirmed the high level of *Ki67* mRNA in AP of *OEx* mice (Fig. 2C). Meanwhile, the up-regulation of cell cycle regulators such as *Ccna2*, *Ccnb1*, *Ccnd1*, *Ccnd2*, *Cdk2*, *Cdk4*, and *Cdk6* were confirmed by qPCR (Fig. 2D). In addition, the immunostaining of apoptosis markers cleaved Caspase-3 showed no difference between the *Ctrl* and *OEx* mice (Fig. 2E). The anti-apoptotic gene *Bcl2* was significantly up-regulated in the AP of *OEx* mice, while apoptotic gene *Bad* was not changed (Fig. 2F). Finally, to investigate whether over-activation of Notch1 signaling causes prostate cancer, we observed the prostates of 1.5-year-old male mice. Compared with the 1.5-year-old *Ctrl* mice, the prostates of *OEx* mice were filled with fluid and unusually edematous, but no cancerous tissue was observed (Fig. 2G&H). Histological staining also confirmed that the prostate was not cancerous in *OEx* mice (Fig. 2G). All these data suggested that the prostates from *OEx* mice underwent enlargement along with age via hyperproliferation.

### The prostate enlargement in *OEx* mice is androgen-dependent

**Fig. 3:**
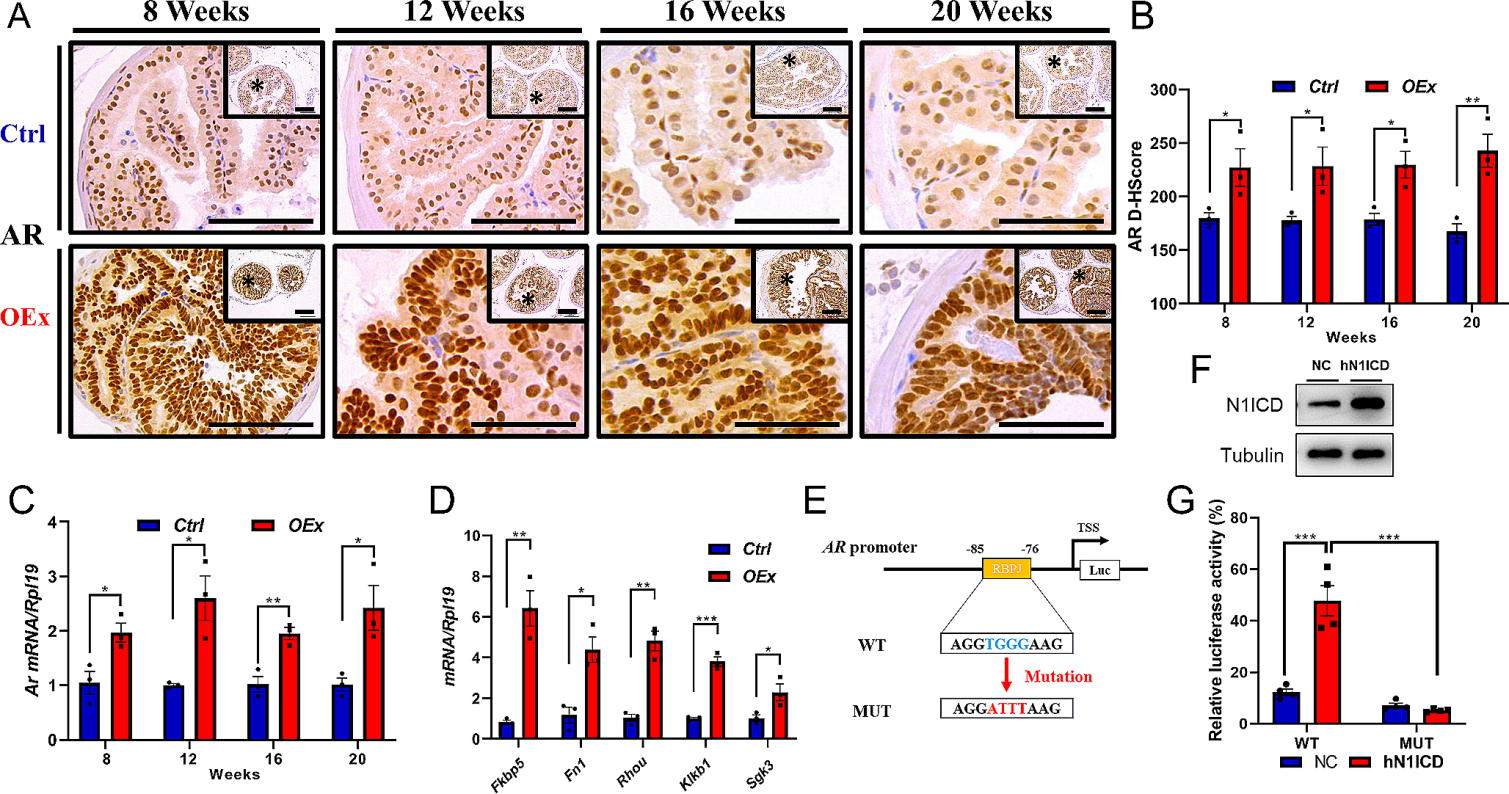
Expression of androgen receptor (AR) in AP from *Ctrl* and *OEx* mice. (**A**) Immunohistochemistry detected AR expression in AP from *Ctrl* and *OEx* mice at 8, 12, 16, and 20 weeks of age. The quantitative expression level of AR protein is shown in (**B**). Data are shown as mean ± SEM, *n* = 3. (**C**) Expression of *Ar* mRNA in AP from *Ctrl* and *OEx* mice at 8, 12, 16, and 20 weeks of age detected by qPCR. Data are shown as mean ± SEM, *n* = 3. (**D**) Expression of AR target genes *Fkbp5*, *Fn1*, *Rhou*, *Klkb1*, and *Sgk3* in AP from *Ctrl* and *OEx* mice detected by qPCR at 20 weeks of age. Data are shown as mean ± SEM, *n* = 3. (**E**) The schematic diagram exhibits RBPJ/AR binding sites and mutation in the AR promoter region. (**F**) Western blot analysis of N1ICD protein level in RWPE1 cells transfected with pcDNA3.1-N1ICD or pcDNA3.1 plasmids. (**G**) Relative luciferase activity of pGL3-AR-WT and pGL3-AR-MUT after overexpression of hN1ICD in human prostatic epithelial cells. Data is shown as mean ± SEM, *n* = 4. Scale bar: 100 μm; D-HScore: digital Histology Score; *P < 0.05; **P < 0.01; ***P < 0.001; ****P < 0.0001

As a male reproductive organ, the development and function of the prostate are mainly controlled by androgen signaling. Androgens bind to the androgen receptor (AR) to promote cell division and proliferation of prostatic epithelial cells [31]. We, therefore, tested if the androgen signaling was altered in *OEx* mice. We first found that the serum testosterone (T) level of *OEx* mice was higher than that of *Ctrl* mice, although the testis morphology showed no difference (Fig. S3). Additionally, immunostaining for AR was significantly higher in AP epithelial cells of *OEx* mice than that of *Ctrl* mice at 8, 12, 16, and 20 weeks of age (Fig. 3A&B). The expression of *AR* mRNA was consistent with AR protein (Fig. 3C). To further verify whether the androgen signaling in AP of *OEx* mice was over-activated, we detected corresponding AR downstream target genes *Fkbp5*, *Fn1*, *Rhou*, *Klkb1*, and *Sgk3*, which were significantly up-regulated in *OEx* mice (Fig. 3D).

To explore the mechanism under which the Notch signaling regulates the transcription of *Ar*, we analyzed the promoter sequence of the *Ar* gene using the JASPAR database and UCSC Genome Browser. We had identified a potential RBPJ binding site at -85 bps before the transcription start site (TSS) of *Ar* gene (Fig. 3E), which is consistent in 35 vertebrates, including humans and mice. To confirm that this binding site is functional, we inserted a 215 bp *AR* promoter sequence into the pGL-3 vector and performed a dual-luciferase assay in prostatic epithelial RWPE1 cells. The results showed that the luciferase activity of the pGL-AR-WT reporter plasmid containing the wildtype sequence was significantly up-regulated by the overexpression of hN1ICD, while as the pGL3-AR-MUT plasmid in which the RBPJ binding site was mutated showed no response to the activated Notch signaling (Fig. 3F&G). These results suggested that *AR* is a direct target gene of canonical Notch signaling.

**Fig. 4:**
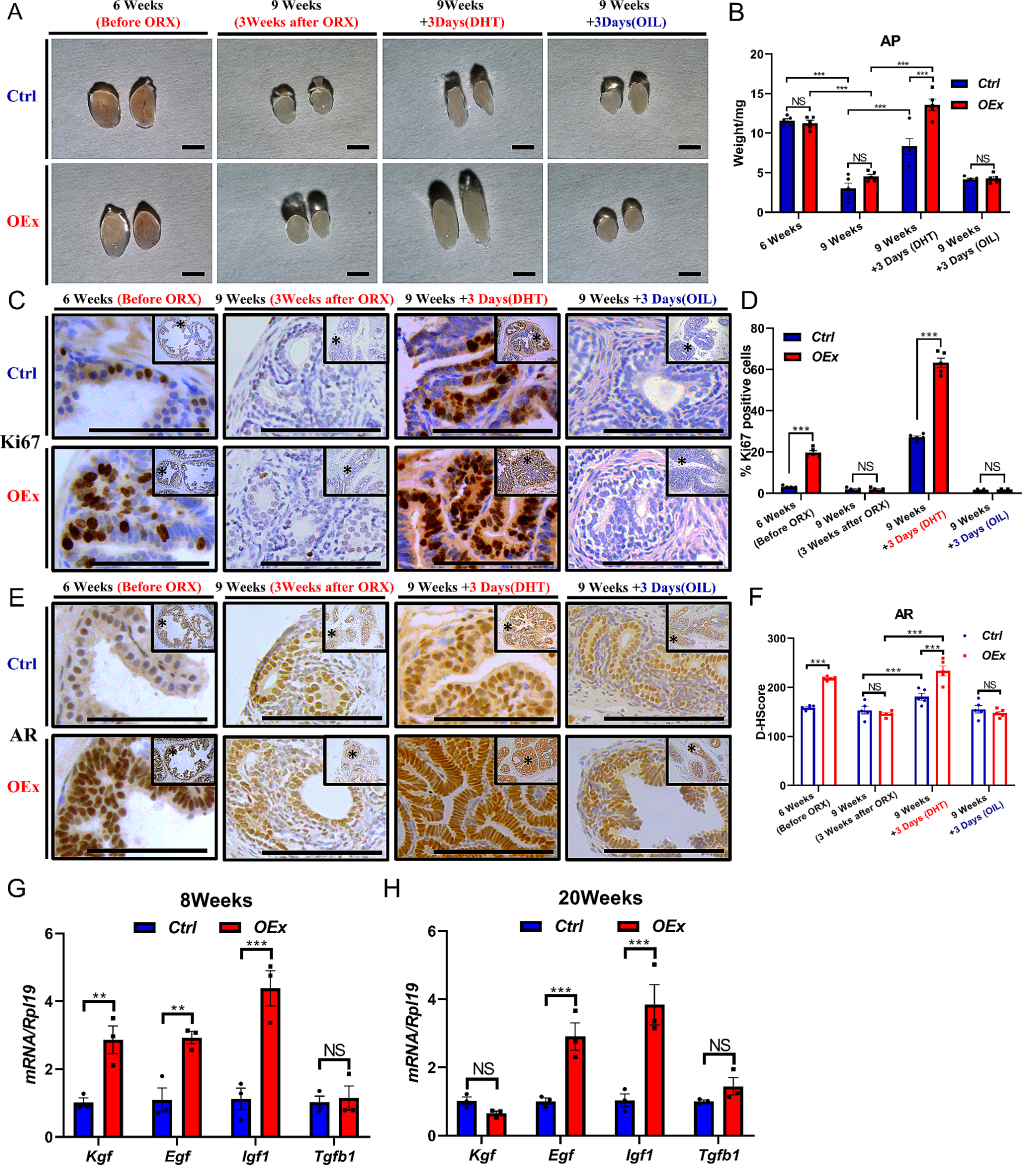
Androgen signaling mediates AP hyperplasia in *OEx* mice. (**A**) AP morphology in *Ctrl* and *OEx* mice before and after orchidectomy (ORX), and treated with DHT or vehicle (OIL) for 3 days. The wet weight of AP is shown in (**B**). Data are shown as mean ± SEM, *n* = 5. (**C**) Immunostaining of Ki67 in AP from *Ctrl* and *OEx* mice before and after ORX, and treated with DHT or OIL for 3 days. The quantitative expression level of Ki67 protein is shown in (**D**). Data are shown as mean ± SEM, *n* = 5. (**E**) AR expression in AP from *Ctrl* and *OEx* mice before and after ORX, and treated with DHT or OIL for 3 days detected by IHC. The quantitative expression level of AR protein is shown in (**F**). Data are shown as mean ± SEM, *n* = 5. (**G**) & (**H**) qPCR detected expression of *Kgf*, *Egf*, *Igf1*, and *Tgfb1* in AP from *Ctrl* and *OEx* mice at 8 and 20 weeks of age. Data are shown as mean ± SEM, *n* = 3. Scale bar: 2 mm in A, 100 μm in C and E; *P < 0.05; **P < 0.01; ***P < 0.001; ****P < 0.0001

To investigate whether the AP hyperproliferation caused by the over-activation of Notch1 signaling was dependent on the activation of androgen signaling, we orchidectomized the mice and supplemented them with or without exogenous androgen (Fig. S4A). 3 weeks after orchidectomy, AP had atrophied, and the average wet weight of *OEx* and *Ctrl* mice were comparable, both decreased from approximately 11 mg to 4 mg (Fig. 4A&B). After 3 days of DHT i.p. injection, AP size and wet weight increased in both *OEx* and *Ctrl* mice. However, the rate of increasing size and wet weight of DHT-treated prostate were significantly larger in the *OEx* mice than that of *Ctrl* mice (Increased by 203.47% v.s 117.89%), indicating that the AP of *OEx* mice grew faster under the stimulation of DHT (Fig. 4A&B). In contrast, in the Oil-treated groups, AP size and wet weight remained low in both *Ctrl* and *OEx* mice without noticeable differences between the two genotypes (Fig. 4A&B). In addition, the histological morphology confirmed the degeneration of AP in *OEx* and *Ctrl* mice after removing endogenous androgen, and the role of DHT in increasing the number of AP epithelial cells between *OEx* and *Ctrl* mice (Fig. S5). The AP epithelial cells of *OEx* mice experienced explosive numbers. The nuclei were squeezed into strips, and it was difficult to distinguish the structures of neighboring cells (Fig. S5). Moreover, Ki67 staining showed that the proliferation of AP epithelial cells in *OEx* mice was significantly stronger than that in *Ctrl* mice with the existence of endogenous androgen or exogenously supplemented DHT, but not when testicles were removed and treated with Oil only, suggesting the existence of androgen was necessary for the hyperplasia phenomena in the *OEx* mice (Fig. 4C&D). On the other hand, we tested the expression level of AR in orchidectomized mice. The result showed that without endogenous androgen, the expression levels of AR are comparable between *OEx* and *Ctrl* mice. However, after 3 days of DHT supplementation, we found that the expression level of AR in *OEx* AP epithelial cells was significantly higher than that of *Ctrl* mice (Fig. 4E&F). These results further reveal that the hyper-proliferation of AP epithelial cells in *OEx* mice is dependent on the activation of androgen signaling.

Studies have shown that the DHT-dependent prostatic enlargement in human BPH is due to the unbalance of downstream paracrine regulators KGF, EGF, and IGFs, which drive the proliferation of epithelial cells, and TGF-β that promotes cell death [5]. In our study, the expressions of *Egf*, and *Igf1* were remarkedly increased in the prostate of *OEx* mice at both 8 and 20 weeks of age compared to the *Ctrl* mice, while *Kgf* was higher expressed at 8 weeks of age only, and *Tgfb* stayed no change (Fig. 4G&H). The increased trends of proliferation-related growth factors and comparable levels of cell death-related growth factors suggested that the imbalance of these regulators might contribute to the DHT-dependent enlargement of the *OEx* prostate.

### Over-activated Notch1 signaling suppresses mitochondrial function and results in excessive accumulation of ROS

**Fig. 5:**
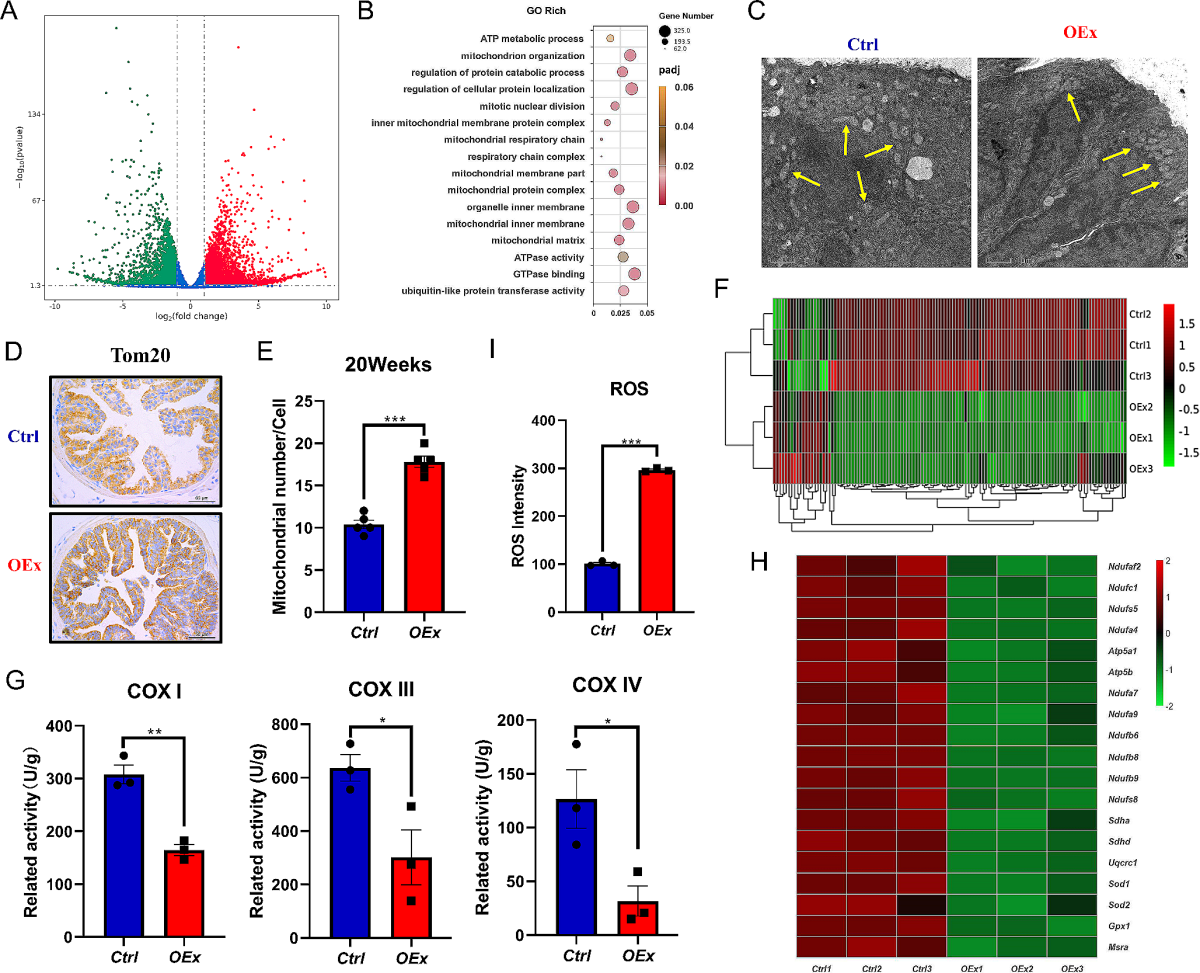
Inhibited mitochondrial functions in AP of *OEx* mice. (**A**) The volcano plot shows differently expressed genes (DEGs) in AP between *Ctrl* and *OEx* mice, detected by RNA-seq. (**B**) GO enrichment analysis DEGs in AP between *Ctrl* and *OEx* mice. (**C**) Transmission electron microscopy images of AP from *Ctrl* and *OEx* mice. (**D**) Immunostaining of Tom20 in AP from *Ctrl* and *OEx* mice at 20 weeks of age. (**E**)The number of mitochondria in AP epithelial cells of AP from *Ctrl* and *OEx* mice at 20 weeks of age. Data are shown as mean ± SEM, *n* = 5. (**F**) Expression of genes regulating mitochondrial function in AP from *Ctrl* and *OEx* mice at 20 weeks of age, from RNA-seq data. (**G**) The activity of prostate mitochondrial complex I, III, and IV in AP from *Ctrl* and *OEx* mice at 20 weeks of age. Data are shown as mean ± SEM, *n* = 3. (**H**) Transcriptional levels of genes involved in mitochondrial complexes ATP production in AP from Ctrl and OEx mice at 20 weeks of age, from RNA-seq data. (**I**) Reactive oxygen species (ROS) intensity in AP from *Ctrl* and *OEx* mice at 20 weeks of age. Data are shown as mean ± SEM, *n* = 3. Scale bar: 1 μm in C, 50 μm in D; *P < 0.05; **P < 0.01; ***P < 0.001

To reveal the effects and regulatory mechanism of Notch1 signaling overactivation on prostate hyper-proliferation, we performed transcriptional profiling using APs from 20-week-old *OEx* and *Ctrl* mice. Comparative analysis of gene expression profiles showed that 8,835 genes were differentially expressed, of which 4,615 were up-regulated, and 3,220 were down-regulated in *OEx* mice prostate (Fig. 5A). Further GO analysis found that many biological functions regarding the mitochondrial function, such as the activities of the mitochondrial protein complex, mitochondrial intimal complex, and mitochondrial respiratory chain were significantly altered (Fig. 5B). Moreover, transmission electron microscopy (TEM) showed that higher in number but smaller in size of mitochondria in the prostate of *OEx* mice compared with *Ctrl* mice. Mitochondria in the *OEx* prostatic epithelial cells were squeezed to the top of the cell (Fig. S6, Fig. 5C&E). The immunostaining showed that Tom20, a component of the mitochondrial outer membrane translocation enzyme located in the mitochondrial outer membrane, was mainly distributed at the top of epithelial cells, which was consistent with the TEM images (Fig. 5D). Lee et al. have shown that canonical Notch signaling induces multiple transcriptional repressors that suppress the transcription of genes encoding mitochondrial complex components [32]. To further investigate whether prostate mitochondria were dysfunctional in *OEx* mice, expression levels of 122 core genes regulating mitochondrial function were analyzed from the RNA-seq data. The results showed that 103/122 genes were markedly down-regulated in the prostate of *OEx* mice (Fig. 5F). Therefore, we next assessed the activity of the mitochondrial complexes. The results showed that the activity of mitochondrial complexes I, III, and IV in the prostate of *OEx* mice was significantly suppressed (Fig. 5G). Transcriptional levels of genes encoding these three mitochondrial complexes and involved in ATP production were also strongly decreased (Fig. 5H). qPCR confirmed the suppression of these genes in the *OEx* mice (Fig. S7A). These results collectively suggested that the mitochondrial function of prostatic epithelial cells is impaired in *OEx* mice, and the increased number of mitochondria might be due to a compensatory mechanism.

The disorder of mitochondrial metabolic activity may bring about the incremental which results in oxidative stress and disrupts various organelles and physiological processes, ultimately destroying the physiological balance [33]. In our study, ROS analysis showed that the intensity of ROS in *OEx* AP was higher than that in *Ctrl* mice (Fig. 5I). Transcription of genes encoding cellular anti-oxidant enzymes was concordant with mitochondrial biogenesis [34]. The expression of anti-oxidant enzymes, such as *Sod1*, *Sod2*, *Gpx1*, and *Msra*, was also significantly inhibited in prostate epithelial cells of *OEx* mice (Fig. S7B, Fig. 5H). Our results indicated that mitochondrial dysfunction led to the decrease of anti-oxidant capacity and increase of ROS level, which might contribute to the hyper-proliferation and enlargement of the prostate in *OEx* mice.

### N-acetyl-L-cysteine alleviates prostatic hyperproliferation in *OEx* mice

**Fig. 6:**
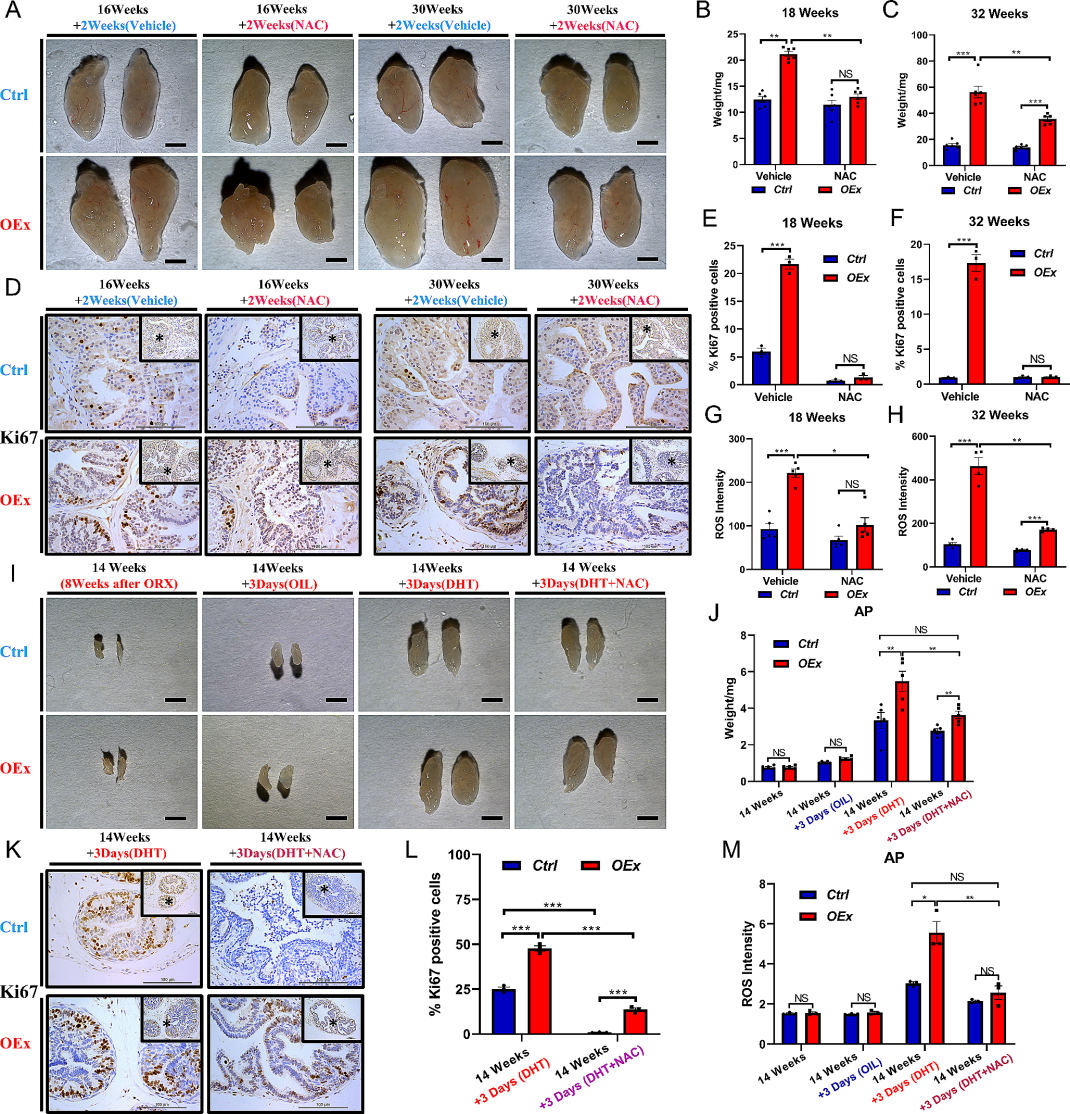
NAC effectively inhibits prostatic hyperplasia in adult *OEx* mice. (**A**) Morphology of *Ctrl* and *OEx* AP treated with N-acetyl-L-cysteine (NAC) or Vehicle for 2 weeks from 16 or 30 weeks of age. The wet weight of APs is shown in (**B**) & (**C**). Data are shown as mean ± SEM, *n* = 5. (**D**) Immunostaining of Ki67 in *Ctrl* and *OEx* AP treated with NAC or Vehicle for 2 weeks from 16 or 30 weeks of age. The quantitative expression level of Ki67 protein is shown in (**E**) & (**F**). Data are shown as mean ± SEM, *n* = 3. (**G**) & (**H**) Prostatic ROS intensity in *Ctrl* and *OEx* AP treated with NAC or Vehicle for 2 weeks from 16 or 30 weeks of age. Data are shown as mean ± SEM, *n* = 5 in G and *n* = 4 in H. (**I**) Mophology of *Ctrl* and *OEx* AP after ORX, treated with OIL, DHT, or DHT together with NAC. The wet weight of APs is shown in (**J**). Data are shown as mean ± SEM, *n* = 5. (**K**) Immunostaining of Ki67 in *Ctrl* and *OEx* AP treated with DHT or DHT together with NAC NAC after ORX. The quantitative expression level of Ki67 protein is shown in (**L**). Data are shown as mean ± SEM, *n* = 3. (**M**) Prostatic ROS intensity in *Ctrl* and *OEx* AP treated with DHT or DHT together with NAC NAC after ORX. Data are shown as mean ± SEM, *n* = 3. Scale bar: 2 mm in A and I, 100 μm in D and K; *P < 0.05; **P < 0.01; ***P < 0.001

To investigate whether the excessive accumulation of ROS caused the hyper-proliferation of prostate in *OEx* mice, we conducted intraperitoneal injection of anti-oxidant N-acetyl-L-cysteine (NAC) in our animal models (Fig. S4B&C). As a result, two weeks of NAC treatment from 16-week-old or 30-week-old significantly reduced the wet weight of AP in *OEx* mice (Fig. 6A). In 16-week-old mice, the younger stage, NAC treatment restored AP the wet weight of *OEx* mice to a comparable level to that of *Ctrl* mice (Fig. 6B). In 30-week-old mice, the older stage, two weeks of NAC treatment could not fully recover the AP wet weight, but still significantly decreased *OEx* induced AP overweight (Fig. 6C). Furthermore, Ki67 staining also showed that AP epithelial cell proliferation significantly decreased in NAC-administrated *OEx* mice compared with vehicle-administrated *OEx* mice at both 16 + 2 and 30 + 2 weeks of age (Fig. 6D-F). Consistent with this, ROS intensity in *OEx* mice was significantly reduced by NAC treatment (Fig. 6G&H). Conversely, 2 weeks of 1.5% H2O2 treatment increased in vivo ROS level, associated with the higher proliferation of prostatic epithelial cells in mice (Fig. S8C-G).

Meanwhile, we conducted NAC treatment in the orchidectomized mouse model. As expected, supplementation of NAC significantly rescued DHT-induced prostatic enlargement in the orchidectomized *OEx* mice, the AP wet weight in this group was similar to that of orchidectomized *Ctrl* mice supplemented with DHT (Fig. 6I&J). Ki67 staining also showed that NAC attenuated AP proliferation in orchidectomized *OEx* mice (Fig. 6K&L). Meanwhile, the ROS intensity of AP in orchidectomized *OEx* mice supplemented with DHT + NAC was also reduced, compared to that of AP in orchidectomized *Ctrl* mice supplemented with DHT (Fig. 6M). However, NAC treatment did not interfere with the expression of AR and Tom20, confirming that the ROS distribution acts as the downstream of androgen signaling and mitochondrial function (Fig. S8A&B-S9A-C). These findings indicated that NAC could alleviate the DHT-dependent prostatic hyperproliferation induced by the over-activation of Notch1 signaling in mice.

### Higher activation of Notch1 signaling in epithelial cells from patients with BPH

**Fig. 7:**
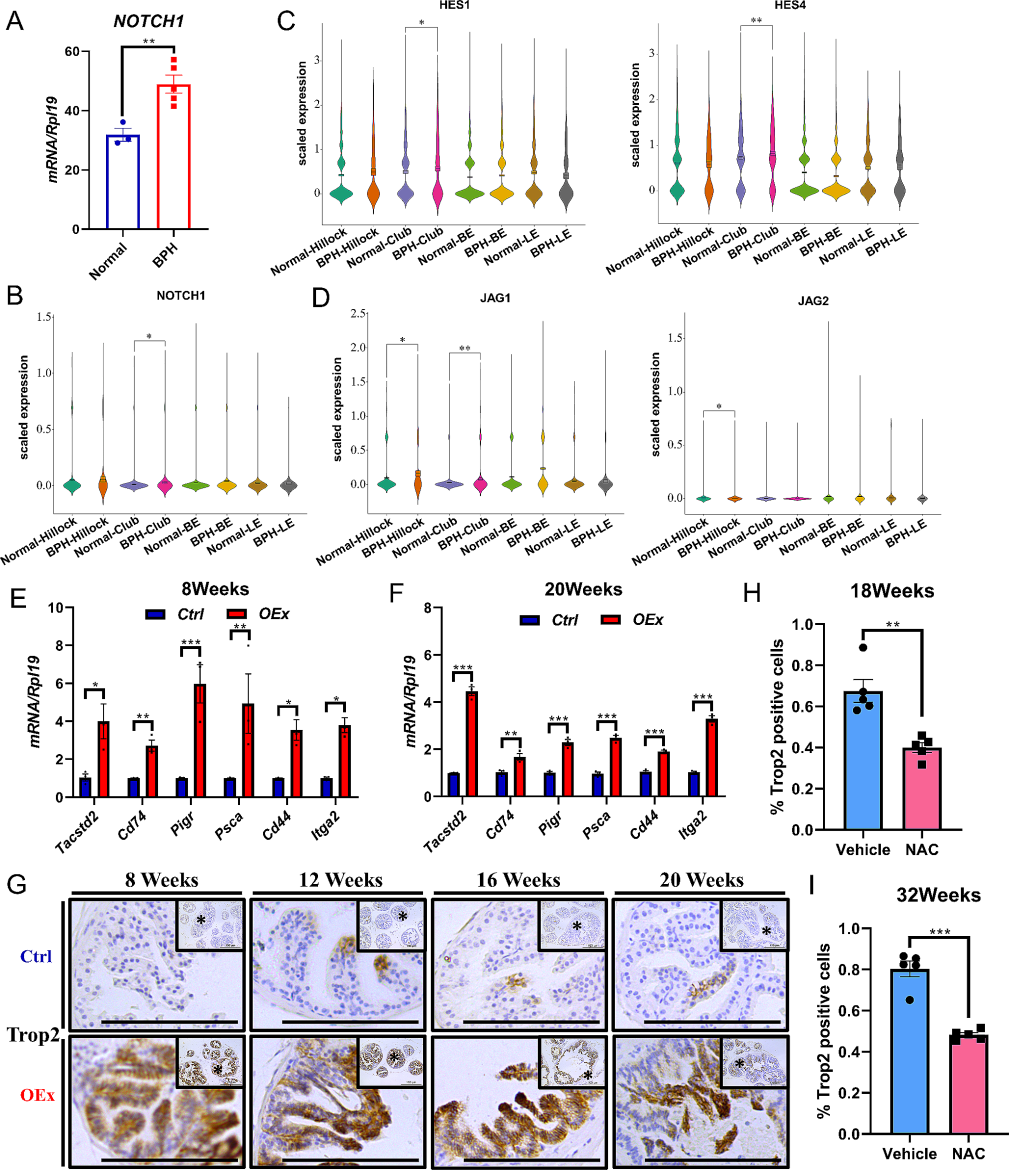
Higher activation of Notch1 signaling in epithelial cells from patients with BPH. (**A**) *NOTCH1* mRNA expression data from benign prostatic hyperplasia (BPH) patients and disease-free controls (Normal). (**B**), (**C**), and (**D**) Violin plot analysis comparing the mRNA levels of *NOTCH1*, *HES1*, *HES4*, *JAG1*, and *JAG2* in epithelial cells from the human prostate with BPH and disease-free controls (Con), from scRNA-Seq data. (**E**) & (**F**) The mRNA expression of *Tacstd2*, *Pigr*, *Psca*, *Cd44*, *Itga2*, and *Cd74* in AP from *Ctrl* and *OEx* mice at 8 and 20 weeks of age. Data are shown as mean ± SEM, *n* = 3. (**G**) TROP2 Immunohistochemistry in AP from *Ctrl* and *OEx* mice at the ages of 8, 12, 16, and 20 weeks. (**H**) & (**I**) The percentage of TROP2^+^ cells in the prostatic epithelial cells from *OEx* mice after two weeks of NAC treatment from 16 and 30 weeks of age. Data are shown as mean ± SEM, *n* = 5. BE: Basal Epithelial Cell; LE: Luminal Epithelial Cell; Scale bar: 100 μm; *P < 0.05; **P < 0.01; ***P < 0.001

We then wondered if Notch signaling over-activation associated hyper-proliferation phenotype occurred in humans. To test this hypothesis, we explored the expression level of *NOTCH1* in human prostates with BPH using online microarray data GSE119195. The result showed that the expression of *NOTCH1* was significantly up-regulated in the prostates from patients with BPH compared with disease-free controls (Fig. 7A). However, our mouse model over-activated Notch signaling in the epithelial cells only but not other cell types. We, therefore, sought to explore the expression level of NOTCH1 in BPH prostate epithelial cells. Dr. Strand and his team have investigated the cellular anatomy of the adult human prostate using single-cell RNA sequencing (scRNA-Seq) and reported two novels discovered epithelial cell types: the urothelial origin club (SCGB1A1^+^) and hillock (KRT13^+^) cells, other than the known luminal and basal epithelial cells from the human prostate, the number of Club cells increases in BPH compared to normal prostate, but Hillock cell number decreases [30, 35]. We then re-analyzed their scRNA-Seq data of prostates from patients with or without BPH. The results suggested that *NOTCH1* mRNA, together with the Notch target genes *HES1* and *HES4*, were significantly higher expressed in the club cells from BPH prostate compared to normal ones, while other Notch receptors showed no difference (Fig. 7B&C; Fig. S10C). Furthermore, the ligand *JAG1* was also up-regulated in the club and hillock cells from the BPH group, suggesting a local over-activation of Notch signaling in these epithelial cells in BPH (Fig. 7D).

To test whether the Club and Hillock-like cells were enriched in the *OEx* mice, we analyzed the expression of gene clusters that representing the Club and Hillock cells in our mouse model. The RNA-seq data showed that many of the genes were highly expressed in the prostates of our *OEx* mice (Fig. S10A). Furthermore, the immunostaining of Club and Hillock cell markers were also higher in the *OEx* mice compared to the Ctrl mice (Fig. S10B). These data suggested that the Club and Hillock-like cells were highly enriched in the prostate epithelial cells of the *OEx* mice.

Club and hillock cells are thought to be prostatic urethra epithelial cells that extend to the glandular prostate, mainly in the proximal transition zone, where BPH is restricted [35]. The number of club cells is increased when BPH occurs [30]. Similar to humans, the TROP2^+^ urethra epithelial cells are found to extend into proximal ducts of the prostate in mice, even though no conventional club epithelial cell type is identified in the mouse prostate [30]. Interestingly, the club and TROP2^+^ cells are both considered potential progenitor cells in the human and mouse prostrates, respectively [36]. They share several characteristic gene expressions, including PIGR and PSCA, which are considered markers of prostate luminal progenitors [35]. The TROP2^+^ epithelial cells can generate large organoids and increase their number in the gland of the large prostate of the 24-month-old mice compared to the small prostate of the 3-month-old ones [37]. Therefore, we tested the expression of these markers in our *OEx* mice. The results showed that the mRNA expression of *Tacstd2* (the gene encodes TROP2), *Pigr*, *Psca*, and other prostatic progenitor markers such as *Cd44*, *Itga2*, *Bcl2*, and *Cd74* were all significantly up-regulated by the over-activation of Notch signaling in the prostate at both 8 and 20 weeks of age (Fig. 7E&F). The immunostaining showed that the number of TROP2^+^ cells was also dramatically increased in the *OEx* mice at all the ages we tested (Fig. 7G). These data suggested that the changes in the number of progenitor cells might contribute to the enlargement of the *OEx* prostate. In addition, three weeks after orchidectomy, the *OEx* mice showed less expression of TROP2, but no significant difference between DHT and OIL groups after 3 days of treatment, suggesting a partial dependence on androgen signaling (Fig. S10D). Moreover, the percentage of TROP2^+^ cells in the prostatic epithelial cells was significantly decreased by two weeks of NAC treatment on 16 and 30-week-old mice, suggesting that Notch signaling induced oxidative stress played a role in controlling the number of these cells (Fig. 7H&I; Fig. S10E).

## Discussion

**Fig. 8:**
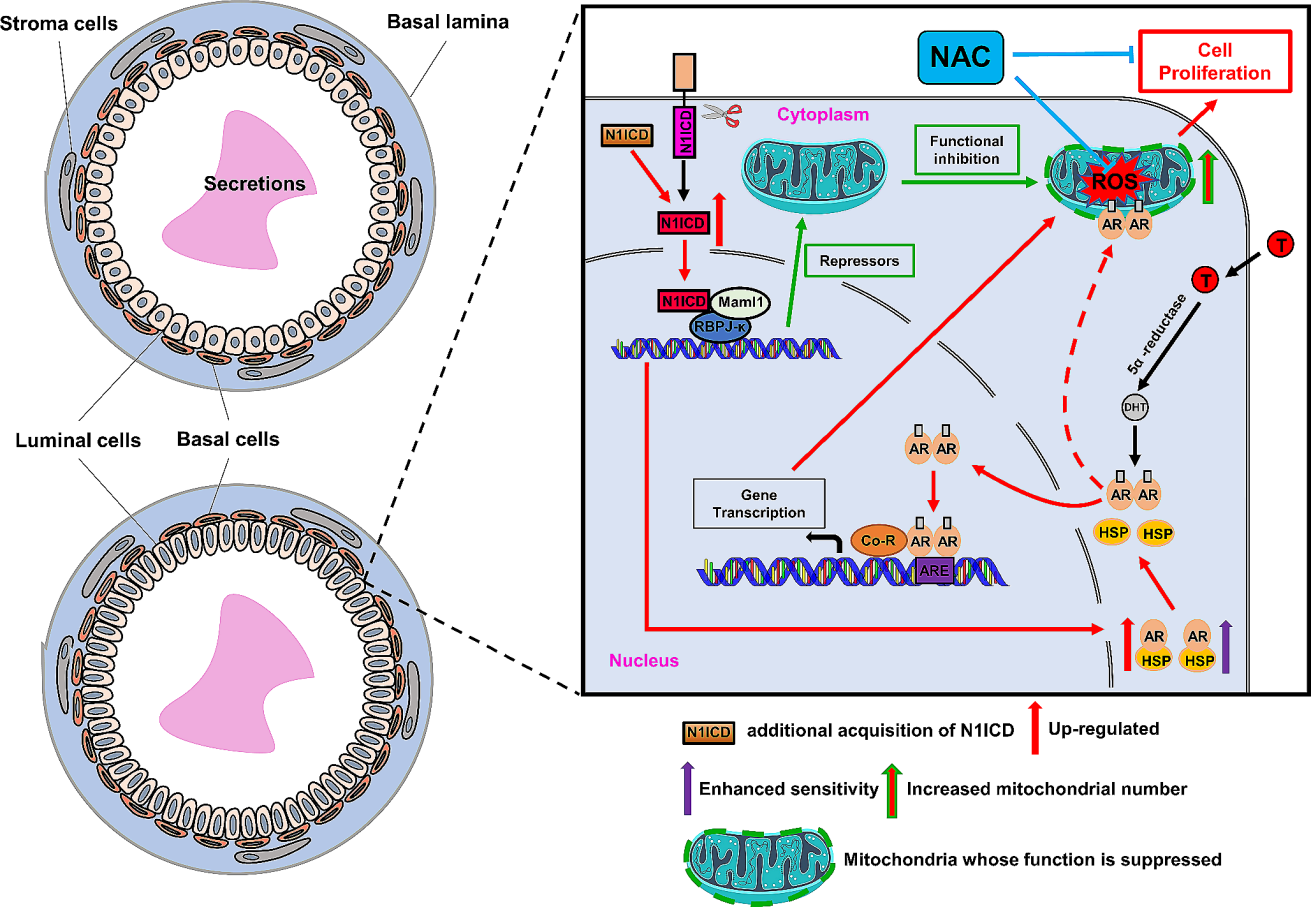
A working model for Notch1 signaling in prostatic hyperplasia. Overactivation of the Canonical Notch1 signaling promotes androgen receptor expression, enhances its sensitivity, and inhibits the Transcription of genes encoding mitochondrial complex components. Under the action of androgen, the activated androgen/androgen receptor signal synergically overactivation of the Canonical Notch1 signaling induces an increased number of mitochondria and an excessive accumulation of ROS, ultimately leading to prostatic hyperplasia. However, NAC can effectively relieve prostate hyperplasia

Both BPH and PCa are considered prostatic diseases related to hyperplasia, age, and inflammation [36, 38–41]. The aberrant regulation of Notch signaling promotes the development, progression, and metastasis of PCa [42]. NOTCH1 is significantly elevated in highly metastatic PCa cell lines [24], metastatic human prostate tissue [43], malignant prostate epithelial cells from primary and metastatic tumors in transgenic mouse models of PCa [23], and bone metastases from human PCa [44]. In this study, we reported an age-related prostate enlargement due to the hyper-proliferation of epithelial cells in a prostatic epithelial specific Notch1 signaling overactivation mice model (Fig. 8). However, the mice do not develop any sign of cancer, even at 1.5-year-old. Aberrant activation of Notch1 signaling alone only leads to hyperplasia and is insufficient to drive prostate carcinogenesis. It has been reported that the loss of PTEN enhances the level of ADAM17, which promotes the activation of Notch signaling, leading to the development of PCa [45]. These findings suggest that cancer formation requires joint action of multiple molecular or pathway components. We, therefore, prefer this phenotype to BPH-like mouse prostate enlargement. The activation of Notch signaling is also found in a subtype of epithelial cells from humans with BPH. In addition, we proved that enlargement mainly depended on the activation of androgen signaling and increased oxidative stress. Furthermore, the increased number of progenitors is also evident in our *OEx* mouse model, in a partially androgen-dependent manner.

Notch signaling has been known as a key regulator in organ development, including the prostate. Many studies have reported that Notch signaling is induced during prostatic development and is necessary for organ growth [46]. Disordered Notch signaling is highly concerned with various hyperplasia diseases in many adult organs, including breast cancer, renal cell carcinoma, lung adenocarcinoma, and endometriosis [22, 47]. In the prostate, the Notch signaling is found to decrease after maturation [46]. Considering the fact that *NOTCH1* expression is significantly higher in human BPH prostates and the enlargement phenotype of the *OEx* mice we provide in this study, we believe that the down-regulation of Notch signaling is necessary, which may result in hyperplasia of the prostate otherwise. An increasing body of studies suggests BPH as an inflammatory disease [38, 48]. Normally, the prostate is considered an immunocompetent organ that hosts a small number of inflammatory cells. However, in BPH patients, more infiltrating T cells, B cells, and macrophages are found, further damaging both epithelial and stromal cells and stimulating cytokine release [49]. In BPH, the high level of IL-17 released from activated T cells stimulates the production of IL-6 by stromal cells up to 9-fold, which further induces the production of FGFs, a key factor that contributes to the stromal growth of the prostate in BPH [50]. In the pathogenesis of endometriosis, an inflammatory gynecological disease in female reproduction, the higher level of IL-6 produced by the macrophages and local epithelial cells leads to up-regulation of NOTCH1 expression and the hyper-proliferation of the ectopic tissue [47]. Therefore, inflammation, in particular the higher level of IL-6, could be a factor that results in the higher level of NOTCH1 in the BPH prostate.

Rapid growth and differentiation of the prostate occur during early development, and benign prostate enlargement is considered a recapitulation of this early embryonic growth [51, 52]. During in-utero growth, epithelial outgrowths from the urethral wall extend outward into the surrounding mesenchyme to form prostate rudiments, which then elongate, branch, and canalize to form a network of ducts that end in acini [53]. Similarly, a reawakening of embryonic growth signals induced new epithelial growth has been proposed for the development of BPH glandular nodules [51, 54, 55]. However, the mechanisms responsible for the activation of peri-urethral glands to proliferative signals during the onset of BPH in middle age remain unknown. Considering the roles of Notch signaling during the early development of the prostate, it is possible that this signaling might contribute to, or be part of, the reawakening of embryonic signaling during prostate enlargement in adults.

As the dominant hormones in male reproduction, androgens are necessary for spermatogenesis, maintenance of sexual capacity, and secondary sexual characteristics [31]. In contrast, serum androgen levels increase significantly during and after puberty, associated with the rapidly increased prostate wet weight [56]. In adults, the size of the prostate shrinks after castration and regrowth after supplementing with androgens [11]. In this study, the prostate enlargement induced by the over-activation of Notch1 signaling is suppressed when the endogenous androgens are removed by orchidectomy and reappear after exogenous DHT treatment, indicating the necessity of androgen for the process. It is generally believed that hormonal disorders, mainly the accumulation of DHT, occurring along with aging may be one of the causes of BPH [57]. Inhibitor of 5α-reductase, the enzyme produces DHT (5ARI), significantly shrinks total prostate volume, and improves BPH-associated LUTS in patients [9]. However, the shrinkage of the prostate is not uniform across the entire gland [58, 59]. Club cells expressing a higher level of NOTCH1 are enriched in men with BPH treated with 5ARI compared to patients not taking 5ARI [30]. In our study, the expression of AR is directly up-regulated by the over-activation of Notch signaling, suggesting a potential mechanism under the 5ARI resistance in clinical treatment, but more straight pieces of evidence are needed. On the other hand, the higher level of Notch signaling only sensitized the androgen signaling but did not bypass it. In addition, It has been reported that overexpressed Jagged1 enhances prostate cancer cell proliferation by interacting with AR, thereby promoting the development of prostate cancer, and silencing of *RBPJ* reduces the expression of the AR and its target genes [60, 61]. Our study further verifies *AR* as a direct downstream target of N1ICD/RBPJ. Furthermore, androgen signaling is reported to be a regulator of Notch signaling via up-regulating its ligands in rat seminiferous epithelium during pubertal development [62] or inducing the expression of Notch1 itself in Sertoli cell lines [63]. Our and others’ data suggest an interaction between the Notch and androgen signaling in the male reproductive system.

The accumulation of harmful ROS levels is one of the byproducts of normal mitochondrial metabolism and homeostasis [64]. The imbalance of mitochondrial function and anti-oxidant defense activity leads to oxidative damage that affects several cellular components, such as lipids, DNA, and proteins [65]. NAC, a free radical scavenger with anti-oxidant and anti-inflammatory activities, is reported to inhibit the proliferation of many cancer cells, including human prostate cancer PC-3 cells. On the other hand, the role of anti-oxidants in preventing BPH has also been reported [66]. Our study proved that the supplementation of anti-oxidant NAC can effectively relieve prostatic hyperplasia in *OEx* mice, indicating that the increase of ROS in *OEx* mice’s prostate mitochondria aggravates the cellular oxidative stress response, which leads to prostatic hyperplasia. This finding partially explains how anti-oxidant medicines and foods may effectively treat BPH in men. Moreover, researchers recently reported that AR transports and concentrates in the mitochondria and plays a crucial role in regulating various mitochondrial functions [67]. Several studies have shown that the production of mitochondrial ATP and the expression of components of respiratory chain complex I, III, and IV are reduced in emasculated rats [68, 69]. Reduction of ATP is observed in granulosa cells from AR null mice [70]. These phenomena are consistent with the observation that the number of mitochondria depends on the presence of androgen. It has also been reported that AR localizes in mitochondria and binds to mitochondrial membrane protein TOM to maintain cell metabolic homeostasis [71]. Therefore, it is highly suspected that AR interacts directly or indirectly with mitochondria to change mitochondrial function and induce prostatic hyperplasia in *OEx* mice.

In conclusion, our study proves that the over-activation of Notch1 signaling in mouse prostatic epithelial cells enhances the adult growth of the organ by increasing its sensitivity to the androgen and inhibiting mitochondrial function. In the presence of androgen, the activated androgen signaling leads to the increase of mitochondria in prostate epithelial cells of *OEx* mice and the excessive accumulation of ROS, which eventually results in prostate hyperplasia. On the other hand, the over-activation of Notch1 signaling also increases the number of TROP2^+^ progenitors, contributing to prostate enlargement. In addition, NAC treatment can effectively reduce prostate hyperplasia in *OEx* mice, which is conducive to developing targeted therapy for the clinical treatment of male prostate hyperplasia.

### Electronic supplementary material

Below is the link to the electronic supplementary material.

Supplementary Material 1

## Data Availability

All data generated or analyzed during this study are available from the corresponding author upon reasonable request.

## Abbreviations

BPH: Benign prostatic hyperplasia
PCa: Prostate cancer
OEx: Overactivation of Notch1 signaling in mice prostatic epithelial cells
AR: Androgen receptor
DHT: Dihydrotestosterone
ROS: Reactive oxygen species
NAC: N-Acetyl-L-Cysteine
