# Supplementary material for: Aberrant activated Notch1 promotes prostate enlargement driven by androgen signaling via disrupting mitochondrial function in mouse
Source: Cell Mol Life Sci. 2024 Mar 28;81(1):155. doi: 10.1007/s00018-024-05143-0 (PMC10973062; doi:10.1007/s00018-024-05143-0)
Supplement: Supplementary file 1 — Supplementary Material 1 [file 18_2024_5143_MOESM1_ESM.pdf]

Figure S1

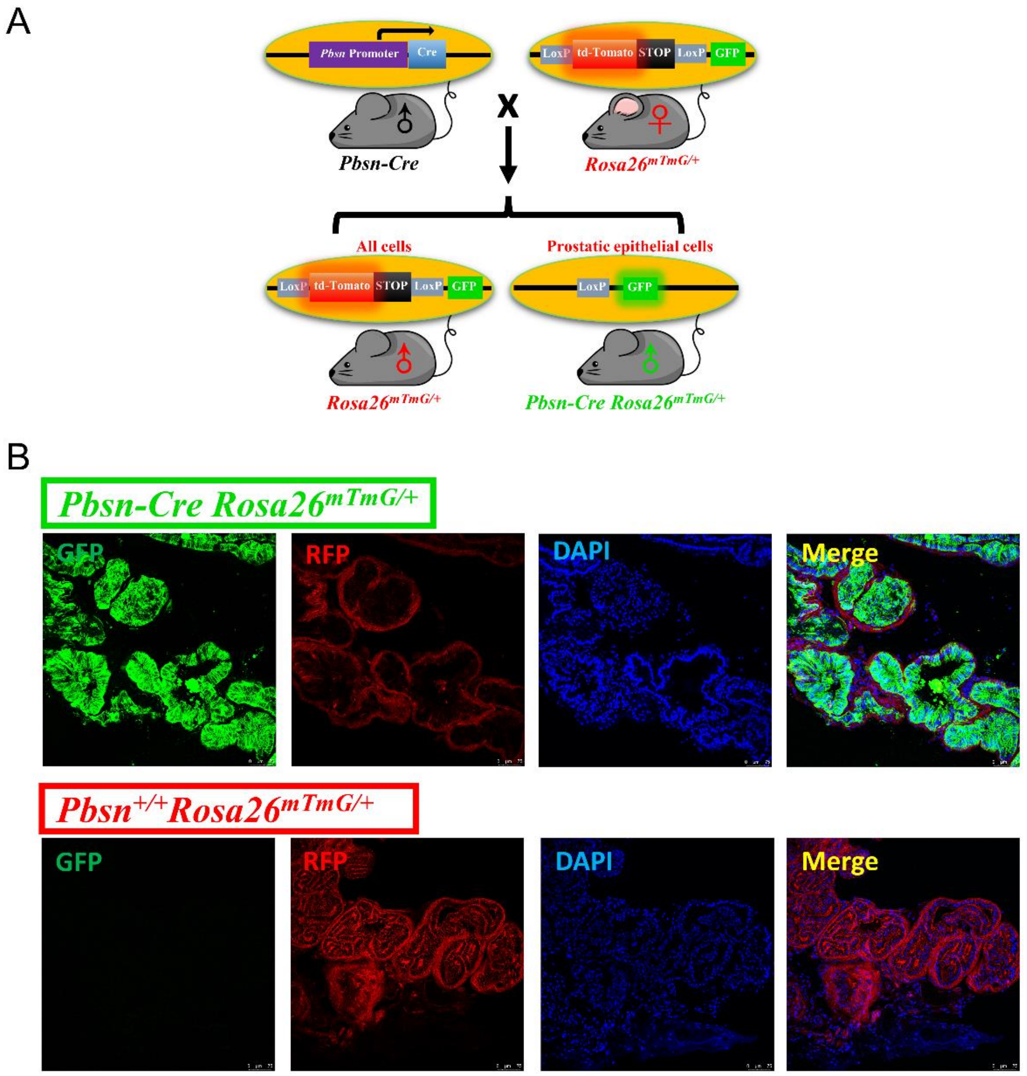

Fig. S1. Generation strategy of *Pbsn-Cre Rosa26*$^{mTmG/+}$ mice. **A** The *Rosa26*$^{mTmG/+}$ and *Pbsn-Cre Rosa26*$^{mTmG/+}$ mice were generated by crossing *Pbsn-Cre* male mice, in which Cre recombinase is driven by *Pbsn* promoter, with *Rosa26*$^{mTmG/mTmG}$ female mice, in which membrane-localized *GFP* is inserted to the Rosa26 locus after a loxP-flanked *td-Tomato* together with transcription termination fragment (STOP). **B** GFP is visible in epithelial cells of AP in *Pbsn-Cre Rosa26*$^{mTmG/+}$ mice but not in *Rosa26*$^{mTmG/+}$ mice at 20 weeks old, confirming that *Pbsn-Cre* has specific activity in prostate epithelial cells. (Scale bar: 75 μm.)

Figure S2

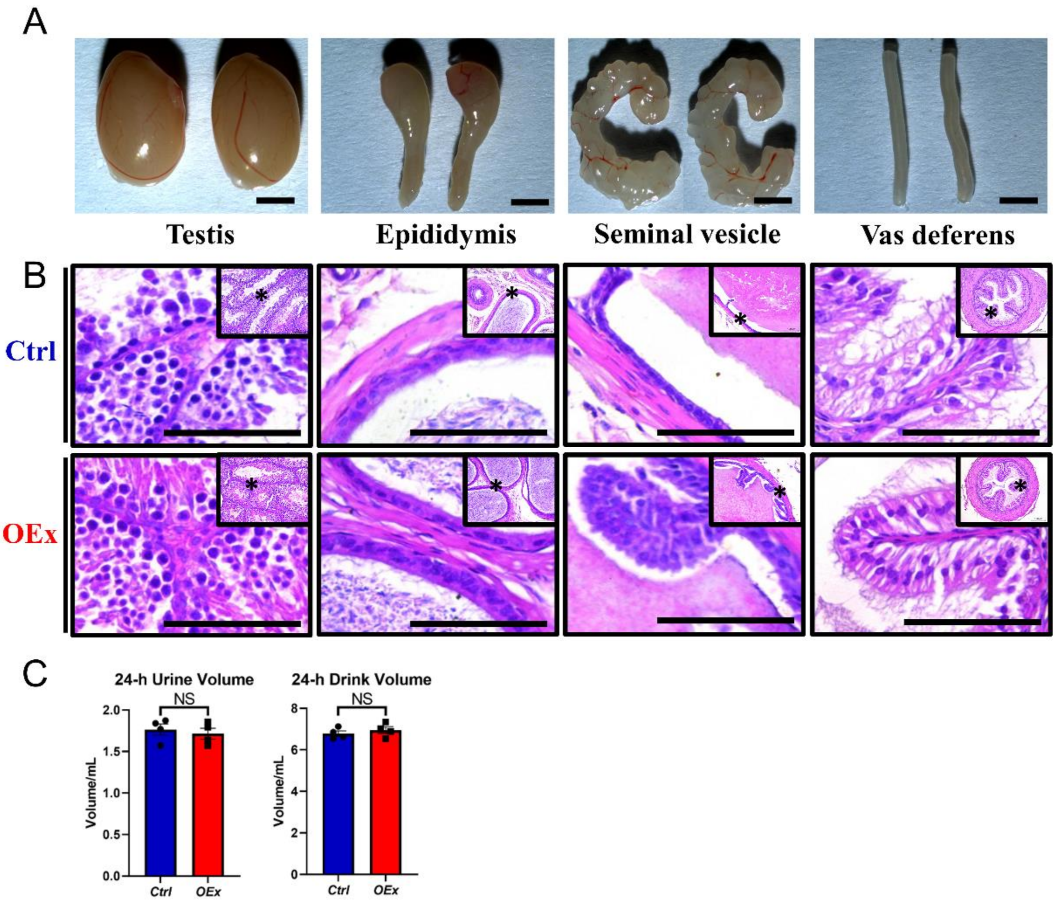

Fig. S2. Morphology of testicle, epididymis, seminal vesicle, and vas deferens in male mice aged 20 weeks. **A** No obvious abnormality was detected in the testis, epididymis, seminal vesicle, and vas deferens of *OEx* mice aged 20 weeks. (Scale bar: 2 mm.) **B** Histological staining shows no abnormal lesions in the testicle, epididymis, seminal vesicles, and vas deferens of *OEx* mice aged 20 weeks. (Scale bar: 100 μm.) **C** Total urine volume output and drink volume show no statistically significant changes between *Ctrl* and *OEx* mice. Data are shown as mean ± SEM, n = 4.

Figure S3

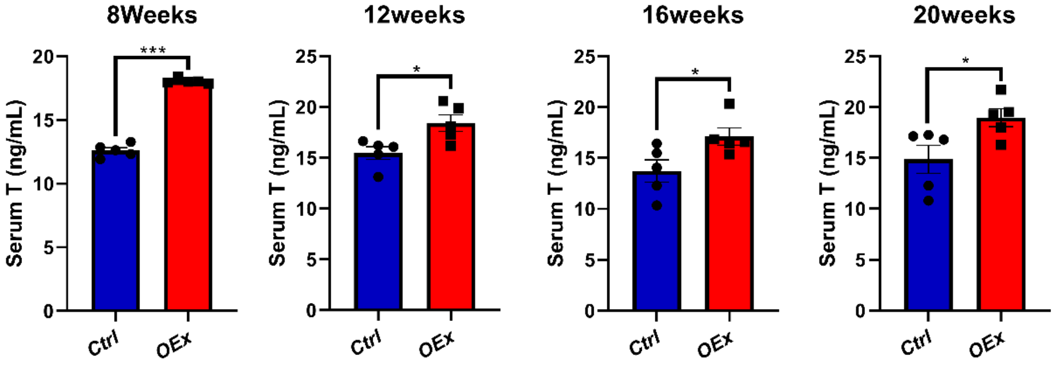

Fig. S3. The serum T level of *OEx* mice was higher than that of *Ctrl* mice at multiple stages. Data are shown as mean ± SEM, n = 5. *P < 0.05, **P < 0.01, ***P < 0.001.

Figure S4

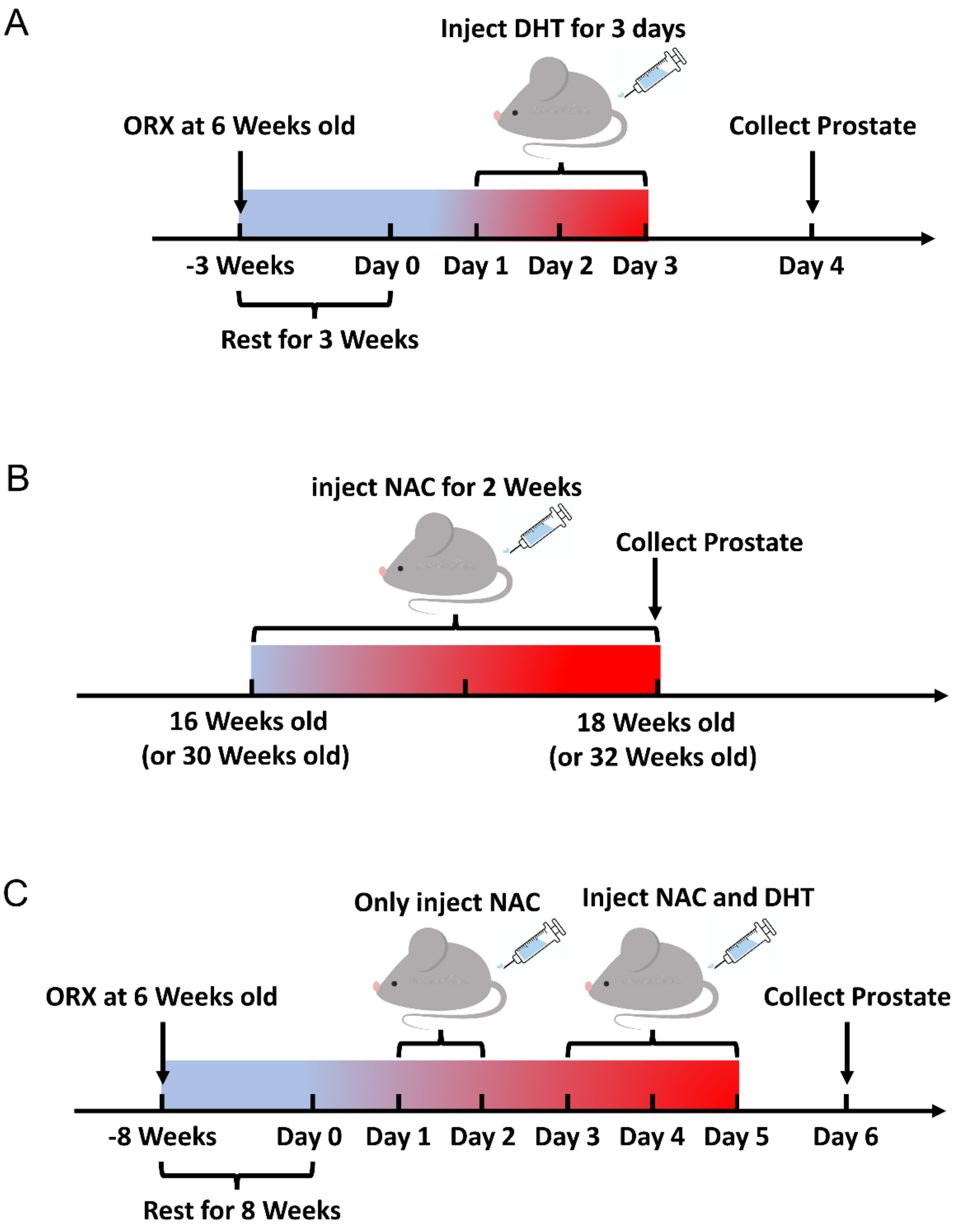

Fig. S4. Diagram of orchidectomy and drug treatment. **A** Experimental schematic diagram of 6-week-old male mice after orchidectomy and resting for 3 weeks, finally continuous subcutaneous injection of DHT for 3 days. **B** Schematic diagram of adult male mice intraperitoneal injection of NAC for two weeks. **C** Experimental schematic diagram of 6-week-old male mice after orchidectomy and resting for 8 weeks, intraperitoneal injection of NAC for 2 days, and then simultaneously injected with DHT and NAC for 3 days. ORX, Orchidectomy; DHT, Dihydrotestosterone; NAC, N-acetyl-L-cysteine.

Figure S5

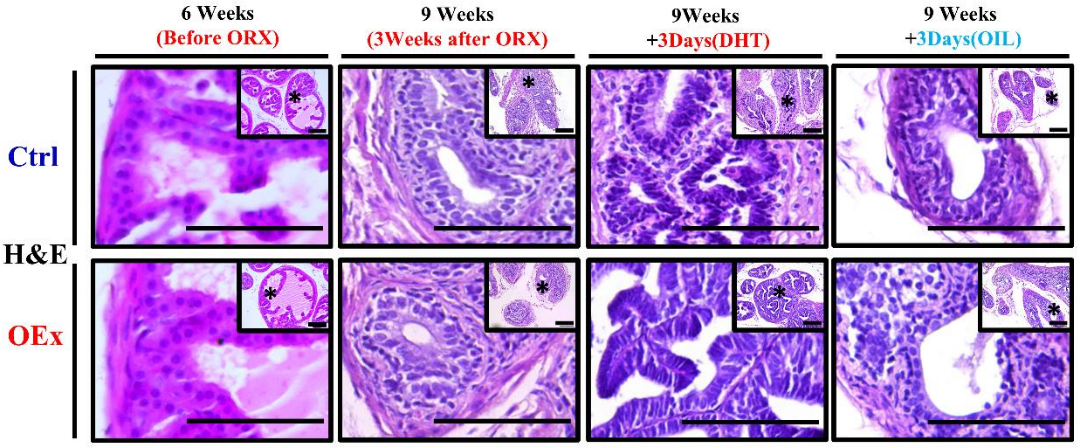

Fig. S5. The proliferation of AP epithelial cells in *OEx* mice was induced by DHT, and the cell morphology was changed. The AP epithelial cells of orchidectomized *OEx* mice proliferated out of control, and the cells were closely packed and severely malformed. ORX, Orchidectomy; DHT, Dihydrotestosterone; OIL, Sesame oil. (Scale bar: 100 μm.)

Figure S6

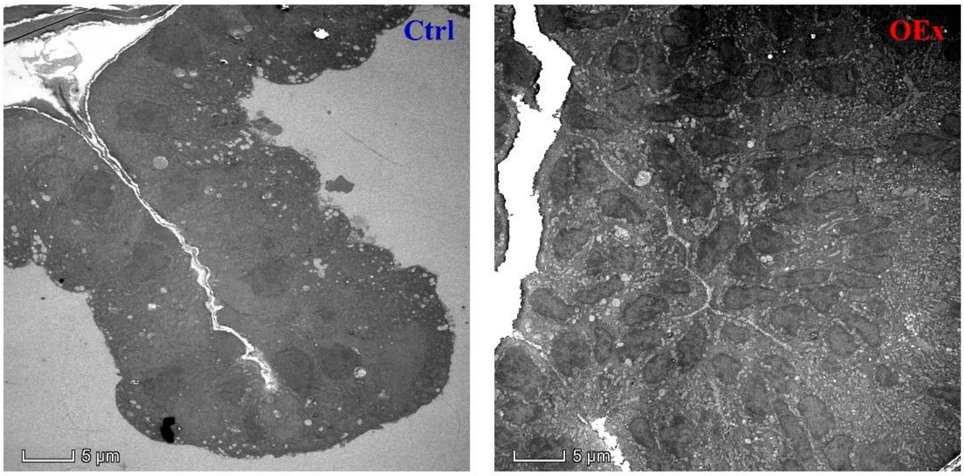

Fig. S6. Transmission electron microscopy of the anterior prostate in a 20-week-old male mouse. The number of mitochondria in the prostate of *OEx* mice was much higher than that of *Ctrl* mice. (Scale bar: 5 μm.)

Figure S7

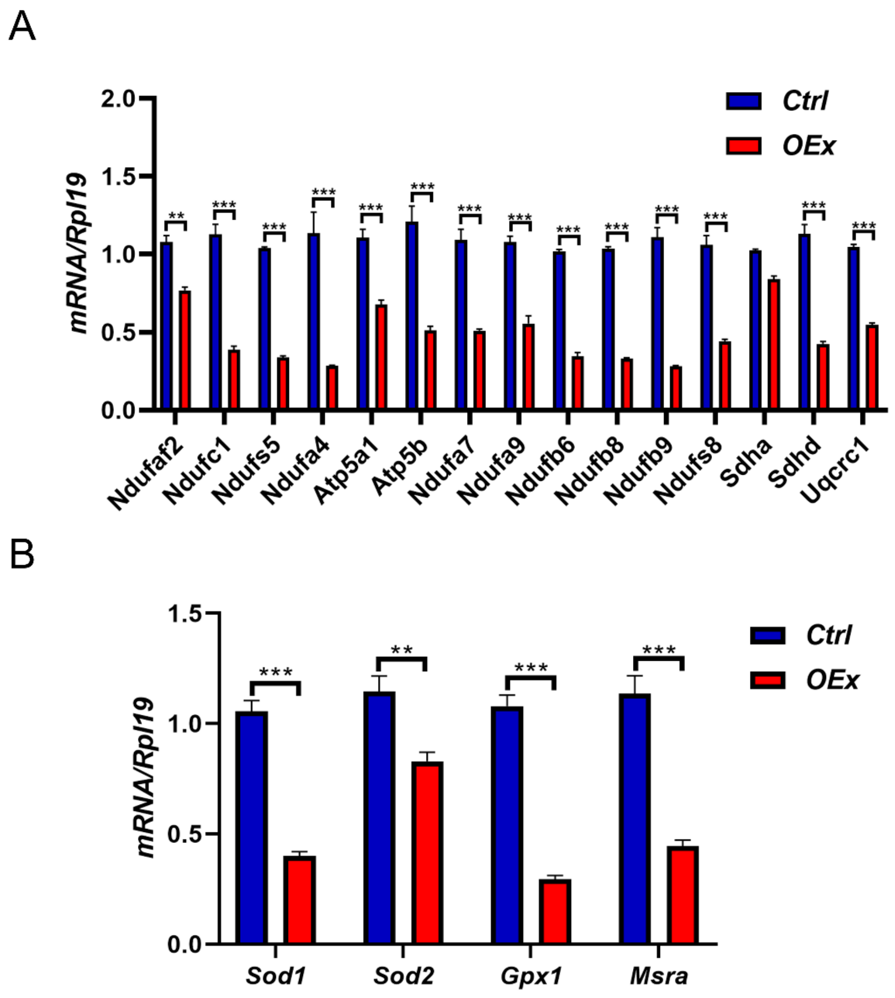

Fig. S7. RT-qPCR experiments confirmed the suppression of those genes encoding three mitochondrial complexes, anti-oxidant enzymes, and involved in ATP production by N1ICD overexpressed. Data are shown as mean ± SEM, n = 3. **P < 0.01, ***P < 0.001.

Figure S8

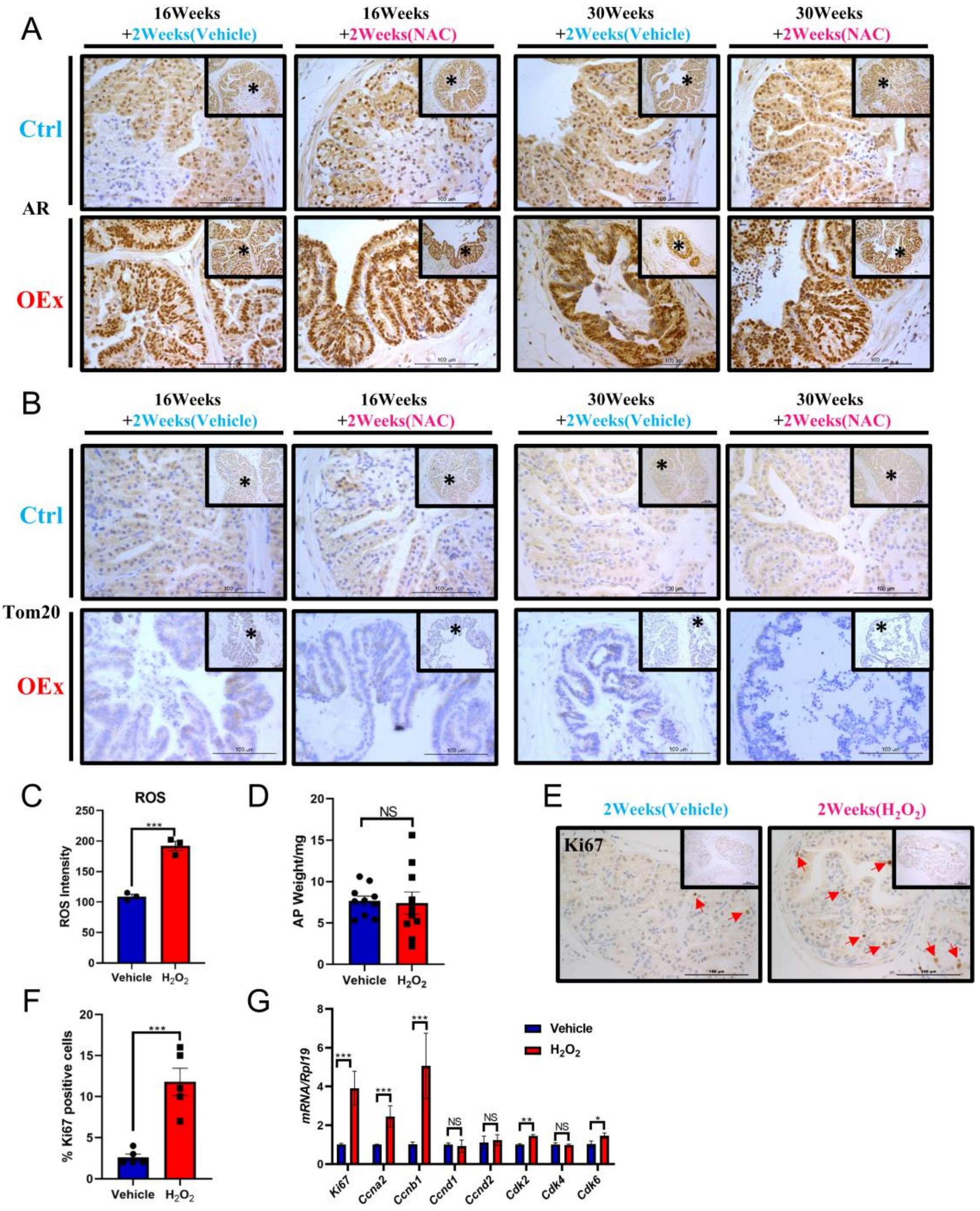

Fig. S8. AR expression pattern and mitochondrial function did not change with NAC treatment. **A** After NAC treatment, the AR expression level in AP epithelial cells of *OEx* mice was still stronger than that of Ctrl mice, and there was no significant difference compared with that of OEx mice without NAC treatment. **B** NAC treatment did not alter the expression patterns of Tom20. NAC, N-acetyl-L-cysteine; AR, androgen receptor; AP, anterior prostate; Tom20, the translocase of the outer mitochondrial membrane 20. (Scale bar: 100 μm.) **C** Reactive oxygen species (ROS) activity in AP from mice with or without H_2O_2 treatment. Mean ± SEM, n = 3. **D** AP wet weight of mice with or without H_2O_2

treatment. Mean ± SEM, n = 5. **E** Immunostaining of Ki67 in AP from mice with or without H_2O_2 treatment. The number of Ki67 positive cells is shown in **F**. Mean ± SEM, n = 5. **G** qPCR detected expression of cell cycle regulators in AP from mice with or without H_2O_2 treatment. Mean ± SEM, n = 3.

Figure S9

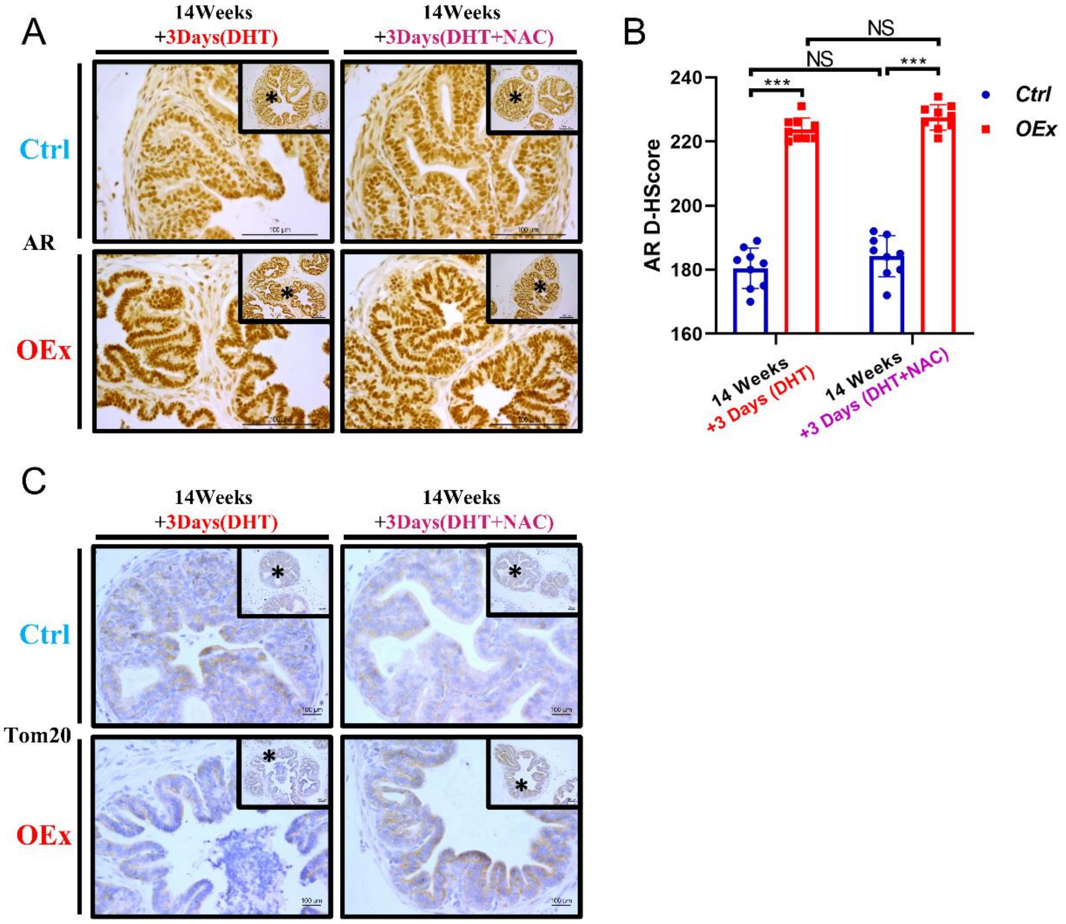

Fig. S9. NAC treatment did not change the expression patterns and function of AR and Tom20 in AP of DHT-induced *OEx* mice. **A** The AR expression level in AP of *OEx* mice treated with DHT and NAC was still higher than that of *Ctrl* mice. The quantitative expression level of AR protein is shown in **B**. Data are shown as mean ± SEM, n = 9. **C** No difference was found in Tom20 expression level in AP with or without NAC treatment. NAC, N-acetyl-L-cysteine; Tom20, the translocase of the outer mitochondrial membrane 20; AP, anterior prostate; DHT, Dihydrotestosterone. (Scale bar: 100 μm.), ***P < 0.001.

Figure S10

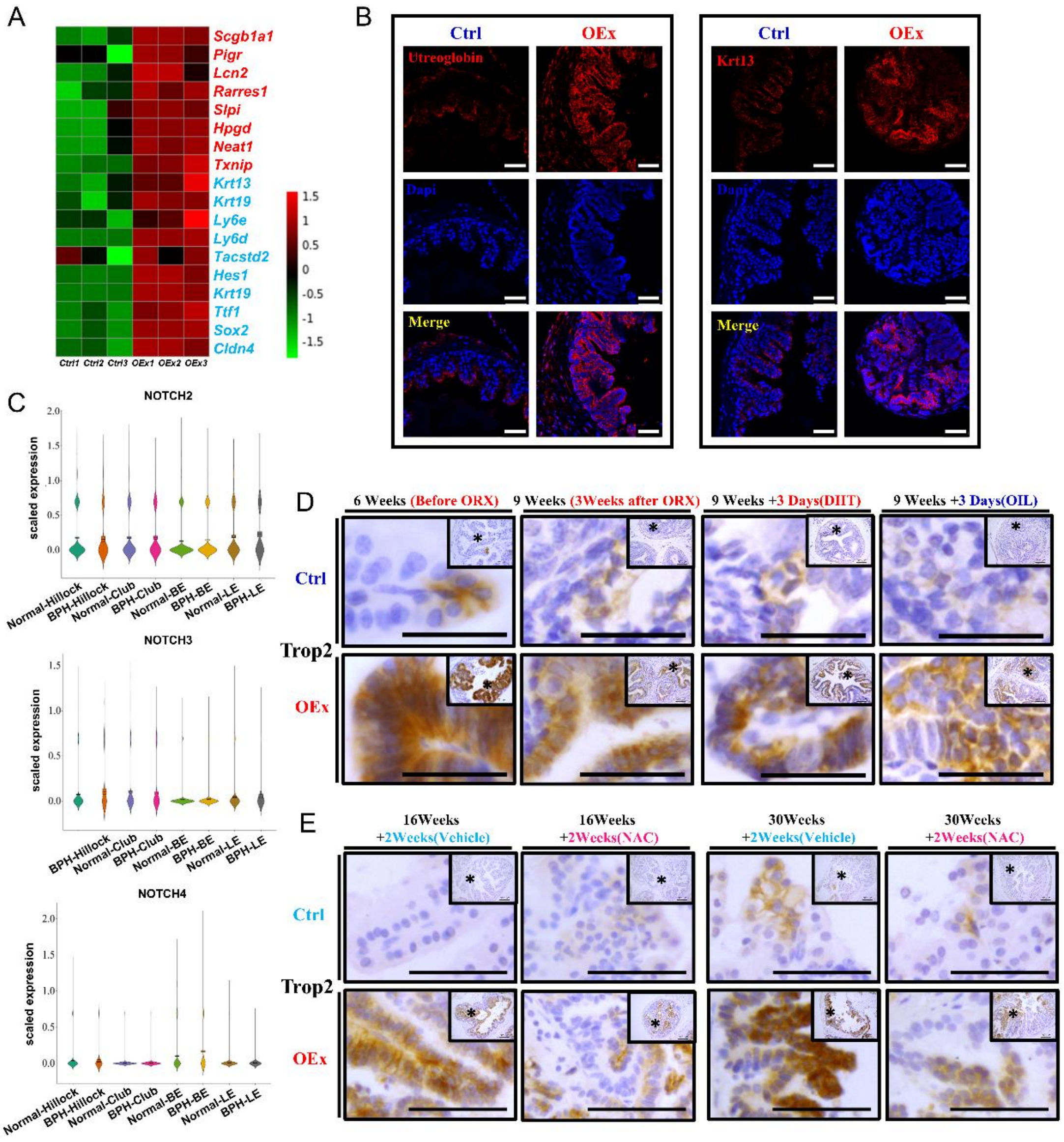

Fig. S10. A Transcriptional clusters of genes representing the Club (Red) and Hillock (Blue) cells in AP of *Ctrl* and *OEx* mice at 20 week-old of age, from RNA-seq data. **B** Immunofluorescence shows that Utreoglobin (encoded by *Scgb1a1*) and Krt13 were highly expressed in AP of *OEx* mice compare to the *Ctrl* mice. (Scale bar: 50 μm) **C** The *NOTCH2*, *NOTCH3*, *NOTCH4* mRNA expression levels showed no difference in the scRNA-Seq data of prostates from patients with or without BPH. **D** Although three weeks after orchidectomy, the *OEx* mice showed less expression of TROP2, no significant difference between DHT and OIL groups after 3 days of treatment. **E** Immunohistochemistry shows that NAC treatment significantly reduced the rate of Trop2 positive cells in the AP tissue of *OEx* mice. NAC, N-acetyl-L-cysteine; AP, anterior prostate. (Scale bar: 100 μm)

Table S1. List of antibodies

Antibody	Type	Manufacturer	Catalog no.	Application
Ki67	Mouse IgG	Santa Cruz	SC-23900	IHC
AR	Rabbit IgG	Santa Cruz	SC-816	IHC
Cleaved-Caspese3	Rabbit IgG	Cell Signaling	9661	IHC
Tom20	Rabbit IgG	Cell Signaling	42406	IHC
Trop2	Rabbit IgG	Abcam	ab214488	IHC
Uteroglobin	Rabbit IgG	Bioss	bs-1487R	IF
Krt13	Rabbit IgG	Abways	CY5744	IF
N1ICD	Rabbit IgG	Abcam	ab128076	WB
TUBULIN	Rabbit IgG	Cell Signaling	2144S	WB

IHC, immunohistochemistry; IF, Immunofluorescence; WB, Western blot.

Table S2. List of primers

Gene	Primer application	Forward sequence	Reverse sequence
Notch1	qPCR	5′-GGAGGTGGATGCTGACTG-3′	5′-TCACTGTTGCCTGTCTCAAG-3′
Egfp	qPCR	5′-AAGCTGACCCTGAAGTTCATCTGC-3′	5′-CTTGTAGTTGCCGTCGTCCTTGAA-3′
Jagged1	qPCR	5′-TAGCCTGTGAGCCTTCCCTGTC-3′	5′-GCAACCGCAGCAATAAGTGAGC-3′
Hey1	qPCR	5′-ACTGTCTCCACCGCTGCTCT-3′	5′-GCTTTCCCCTCCCTTGTTCT-3′
Hes5	qPCR	5′-GAGAAGATGCGTCGGGACC-3′	5′-GGCGAAGGCTTTGCTGTG-3′
Rbpj	qPCR	5′-GCCTGTTGTGACAGGGAAGT-3′	5′-GCACTGTTTGATCCCCTCGT-3′
Ki67	qPCR	5′-CCAGCTGCCTGTAGTGTCAA-3′	5′-CCATGTCTCAGCCTCACAGG-3′
Cyclin A2	qPCR	5′-GTCAACCCCGAAAAACTGGC-3′	5′-TTAAGAGGAGCAACCCGTCG-3′
Cyclin B1	qPCR	5′-CTCTCCAAGCCCGATGGAAA-3′	5′-CTGCGTCTACGTCACTCACT-3′
CyclinD1	qPCR	5′-CCTCTCCTGCTACCGCACAA-3′	5′-GAGATGGAGGGGGTCCTTGTT-3′
CyclinD2	qPCR	5′-GCCAAGATCACCCACACTGA-3′	5′-GCGTTATGCTGCTCTTGACG-3′
CDK2	qPCR	5′-GGAGAAGATTGGAGAGGGCAC-3′	5′-GCCACAACTTCTCCCGTCAA-3′
CDK4	qPCR	5′-GTGGCCCTCAAGAGTGTGAG-3′	5′-CACAGACATCCATCAGCCGT-3′
CDK6	qPCR	5′-TCCTGCTCCAGTCCAGCTAT-3′	5′-CCACGTCTGAACTTCCACGA-3′
Bad	qPCR	5′-CTTGAGGAAGTCCGATCCCG-3′	5′-GCTCACTCGGCTCAAACTCT-3′
Bcl2	qPCR	5′-GCGTCAACAGGGAGATGTCA-3′	5′-GCATGCTGGGGCCATATAGT-3′
Ar	qPCR	5′-CCCTGAGGCCGCTAACATAG-3′	5′-GGGCTTGAGGAGAACCATCC-3′
Fkbp5	qPCR	5′-TATGCTTATGGCTCGGCTGG-3′	5′-GAGTATCCCTCGCCTTTCCG-3′
Fn1	qPCR	5′-CCCCAACTGGTTACCCTTCC-3′	5′-GGTTGGTGATGAAGGGGGTC-3′
Rhou	qPCR	5′-CAGCTACACCACTAACGGCT-3′	5′-CCATCTACAGACACCACGGC-3′
Klkb1	qPCR	5′-TGGCTCCCCAACTAGGATCA-3′	5′-AGCGTTTGTTCCTCCCACAA-3′
Sgk3	qPCR	5′-TTTCCTGAACCCAGAGCGAG-3′	5′-TTGATGGAGTGCAGGTAGCC-3′
Rpl19	qPCR	5′-TCATGGAGCACATCCACAAGCTGA-3′	5′-CGCTTTCGTGCTTCCTTGGTCTTA-3′
Sod1	qPCR	5′-GGAACCATCCACTTCGAGCA-3′	5′-CCCATGCTGGCCTTCAGTTA-3′

Sod2	qPCR	5′-GCCTGCTCTAATCAGGACCC-3′	5′-GTAGTAAGCGTGCTCCCACA-3′
Gpx1	qPCR	5′-TCAGTTCGGACACCAGAATGG-3′	5′-GGAAGGTAAAGAGCGGGTGA-3′
Msra	qPCR	5′-CGGTCAGCAGTCTATCCCAC-3′	5′-CTTGTCCCTCTCGGATGTCG-3′
Tacstd2	qPCR	5′-ACAACGATGGCCTCTACGAC-3′	5′-TTTGGTCTCCCTTGTCCGTG-3′
Psca	qPCR	5′-CAGTTGCTTTACATCGCGCA-3′	5′-CAGGTCAGAGTAGCAGCACG-3′
Cd74	qPCR	5′-TGGATGGCGTGAACTGGAAG-3′	5′-TTCTTCCTGGCACTTGGTCA-3′
Pigr	qPCR	5′-AGAACTCCAGGTTGCCGAAG-3′	5′-ACGGATAGTGGCAGGAAACG-3′
Cd44	qPCR	5′-CCTTGGCCACCACTCCTAAT-3′	5′-TCCGTTCTGAAACCACGTCT-3′
Itga2	qPCR	5′-TGGTAGTTGTGACCGATGGC-3′	5′-CTGCTATGCCGAACCTCAGT-3′
Ndufaf2	qPCR	5′-GCCCCGTATTTTGGAAGGGA-3′	5′-CAGGACCCTGGCTGAAAAGT-3′
Ndufc1	qPCR	5′-GTAGTGCTGCGCTCGTTTTC-3′	5′-TTCGACCGTGTTGAAGAGCA-3′
Ndufs5	qPCR	5′-CTATAAGAACGCCGCTCGGT-3′	5′-CTTGCACTCCTTTTTCGCCC-3′
Ndufa4	qPCR	5′-GTATGTGATGCGCTTGGCAC-3′	5′-TGTTCCATGGCTCTGGGTTG-3′
Atp5a1	qPCR	5′-ACTGCATCTACGTCGCGATT-3′	5′-CGCATCCGTCAGTCTCTTCA-3′
Atp5b	qPCR	5′-AGCTCTGACTGGTTTGACCG-3′	5′-GCCCAATAAGGCAGACACCT-3′
Ndufa7	qPCR	5′-CCCAGTCACAAGCTGTCCAA-3′	5′-TTGAGGGAGGCACAACTTCC-3′
Ndufa9	qPCR	5′-TGAAGCAGCCGGTGTATGTT-3′	5′-AAGAGCAGGTACCGGTTTGG-3′
Ndufb6	qPCR	5′-GGAGCTAAGGAGACGATGGC-3′	5′-ACCATGTTCTTCCACACGGC-3′
Ndufb8	qPCR	5′-GGTATGGCGACTACCCGATG-3′	5′-GACGTGTCCACACGATTCCT-3′
Ndufb9	qPCR	5′-GGCATCCCTCTGAGAAAGCAA-3′	5′-CTTAACCTCCCGATCCCAGC-3′
Ndufs8	qPCR	5′-TGGCGGCAACGTACAAGTAT-3′	5′-CTTAGGGTCATGCCCAGTCC-3′
Sdha	qPCR	5′-TCATCTGCCTCCTGAGCAAC-3′	5′-ACTGGGATGGGCTCCTTAGT-3′
Sdhd	qPCR	5′-GACCCGCTTATGTGTCAGCA-3′	5′-AGTGGAGAGATGCAGCCTTG-3′
Uqcrc1	qPCR	5′-GGCTAACCCAGACAACGTGA-3′	5′-TGTTAGCCACAGCAACGGAA-3′

N1ICD, Notch1 intracellular domain; *Hey1*, hairy enhancer of split-related 1; *Hes5*, hairy enhancer of split 5.
